# Information on latent tuberculosis infection in higher education: social representations of nursing students*

**DOI:** 10.1590/1980-220X-REEUSP-2025-0029en

**Published:** 2025-09-08

**Authors:** Erlon Gabriel Rego de Andrade, Ivaneide Leal Ataíde Rodrigues, Laura Maria Vidal Nogueira, Eliza Paixão da Silva, Adriely Alciany Miranda dos Santos, Letícia Gabriela Noronha Rodrigues, Débora de Cássia Quaresma Silva, Vitória de Cássia Quaresma Silva

**Affiliations:** 1 Universidade do Estado do Pará, Escola de Enfermagem Magalhães Barata, Programa de Pós-Graduação em Enfermagem, Belém, PA, Brazil.; 2 Universidade do Estado do Pará, Centro de Ciências Biológicas e da Saúde, Escola de Enfermagem Magalhães Barata, Belém, PA, Brazil.; 3 Universidade Federal do Pará, Instituto de Ciências da Saúde, Faculdade de Enfermagem, Belém, PA, Brazil.

**Keywords:** Latent Tuberculosis, Students, Nursing, Universities, Social Representation, Psychology, Social

## Abstract

**Objective:**

To analyze the social representations of nursing students regarding the sharing of information about latent tuberculosis infection in higher education.

**Method:**

Descriptive, qualitative study, anchored in the procedural aspect of the Theory of Social Representations. It was carried out with 37 students who reacted to the tuberculin test, enrolled from the 1st to the 5th grade, in the Undergraduate Nursing Course at a public university in Belém, Pará, Brazil. Between March and July 2023, semi-structured individual interviews were conducted, the *corpus* of which was subjected to lexical analysis with the software *Interface de R pour les Analyses Multidimensionnelles de Textes et de Questionnaires* (0.7, alpha 2), using descending hierarchical classification.

**Results:**

A total of 1,939 text segments were identified, of which 1,686 (86.95%) were used, generating seven lexical classes. It was decided to detail class 7, which outlined two representational sets, addressing how information was shared and suggestions on the topic.

**Conclusion:**

The limitations that weakened the timely sharing and quality of information motivated students to make suggestions to solve the challenges inherent to this context or mitigate its harmful effects.

## INTRODUCTION

Currently, tuberculosis (TB) still represents an important public health problem, due to the occurrence of pulmonary and extrapulmonary forms, respectively characterized by the involvement of the lungs and other anatomical and physiological structures, marked by the multiplication and spread of the *Mycobacterium tuberculosis*. Another concern is the state that precedes the illness, known as latent tuberculosis infection (LTBI), which results from exposure to the bacillus expelled by people with untreated pulmonary TB or laryngeal TB and is characterized by its installation in the lungs, generating an adaptive immune response, without triggering signs and symptoms, with no possibility of transmission^(1,2)^.

Living with the bacillus, immunocompetent people may never develop TB, but evidence shows that the greatest risk of illness occurs in the first two years after infection, especially among groups with immunological vulnerability, such as those under the age of two or over 60, those using immunosuppressive therapy, those living with the human immunodeficiency virus (HIV), those with diabetes mellitus and/or other chronic non-communicable diseases^(2)^. Estimates indicate that LTBI affects around a quarter of the global population, representing a large contingent of susceptible individuals^(3)^.

Provided by the Brazilian Public Health System (*Sistema Único de Saúde* – SUS), the tuberculin skin test is the predominant test for diagnosing LTBI in Brazil. It requires a qualified professional to apply 0.1 ml of tuberculin, intradermally, to the middle third of the anterior surface of the forearm, generating a cutaneous hypersensitivity reaction, the induration of which must be measured, considering the largest transverse diameter. A millimeter ruler is used to read and interpret the test between 48 and 72, or up to 96 hours, after application, and the result must be recorded in millimeters (mm), even when there is no induration (zero mm), with an individual with an induration equal to or greater than five millimeters being considered a reactor (infected)^(4)^.

To develop effective control strategies, aligned with epidemiological surveillance, it is necessary to investigate LTBI, especially in groups characterized by greater exposure to the bacillus, such as students in health areas, among which nursing students stand out, as studies have already revealed a high prevalence of infection in this population^(5,6)^. This exposure occurs because, during the course, these students are included in supervised practical activities, such as those carried out in Primary Health Care (PHC), through collective health programs, with different human groups, highlighting people with TB and their companions, caregivers, and family members^(7)^.

In addition to clinical-epidemiological aspects being relevant to understanding the topic, it is understood that psychosocial aspects permeate students’ experiences in situations of exposure and possible contagion, since, once infected and aware of their condition based on diagnosis, students can change the way they understand their reality and the relationships woven within it with other actors, such as users of health services, internalizing the need to undertake or transform self-care practices, to avoid illness, and care practices directed at human groups, to meet their biopsychosocial needs. Therefore, in higher education, timely access to quality information on LTBI, during university training, can favor such practices.

This points to the Theory of Social Representations (TSR), originating from social psychology and formulated by Serge Moscovici in the 1960s. It aims to understand social representations (SR), a form of knowledge that guides strategies with which individuals communicate, interact, and behave in society^(8)^. SRs establish a common language, which distinguishes groups according to their social affiliations, that is, characteristics that indicate differences in the conditions of production of SRs^(9)^.

They have three basic dimensions: information, defined by knowledge about the psychosocial object and acquired through experiences; attitude, marked by the judgment that the individual makes about the object, inducing himher to a symbolic position; and field of representation or dimension of the image, which idealizes a social model, with representational elements arranged in an orderly and hierarchical manner. Therefore, information plays a crucial role in the production of SR and helps to understand everyday practices^(8)^.

Given the relevance of the topic, the guiding question was developed: how do nursing students represent the sharing of information about LTBI in higher education? To answer this question, this study aimed to analyze the social representations of nursing students regarding the sharing of information about latent tuberculosis infection in higher education.

## METHOD

### Design of Study

Descriptive study, with a qualitative approach, anchored in the procedural aspect of TSR and guided by the instrument Consolidated Criteria for Reporting Qualitative Research (COREQ), considering its 32 items, distributed in three domains, inherent to the research team, the study design, and the analysis and presentation of the results^(10)^. In line with this approach, the procedural aspect of TSR allows understanding the process of constructing SR, revealing its constituent elements, which can be identified, for example, through the expression of cognitive, ideological, imagery and informational aspects, or which denote attitudes, beliefs, opinions, and social values^(9)^.

### Local

It took place in the Nursing Undergraduate Course at a public university in the city of Belém, state of Pará, Brazil. With a duration of five years, the course is operationalized in five series and 10 semester periods (each grade consisting of two blocks, identified as block I and block II), totaling 5,000 hours of theoretical and practical activities^(11)^. This scenario was chosen because its characteristics meet the interest of investigating the topic in the daily lives of nursing students.

### Population and Selection Criteria

Thirty-seven students who reacted to the tuberculin test, with a result equal to or greater than five millimeters, regularly enrolled, and studying from the 1st to the 5th grade, participated. The students were previously tested as part of the activities of a multicenter research project, developed within the scope of the National Academic Cooperation Program (*Programa Nacional de Cooperação Acadêmica* – PROCAD), provided by the Coordination for the Improvement of Higher Education Personnel (*Coordenação de Aperfeiçoamento de Pessoal de Nível Superior* – CAPES/Ministry of Education) to stimulate collaborative networks between Master’s and Doctorate Postgraduate Programs, including Postgraduate Programs in Nursing. This research involved four public higher education institutions (HEIs), located in the capitals of four Brazilian states: Belém (Pará), Manaus (Amazonas), Rio de Janeiro (Rio de Janeiro), and Terezinha (Piauí).

In Belém, testing took place between December 2022 and February 2023, a period in which there were approximately 345 students enrolled in the course. A total of 269 (77.97% adherence) were tested, with 74 reactors (27.51% of those tested), 37 (50.00%) of them being included in this study. This number met the sample recommendation of 20 to 30 participants, indicated in the literature as empirically satisfactory for achieving objectives in qualitative research, and considered data saturation, evidenced when these were shown to be sufficient and, therefore, additions would not change the authors’ understanding of the psychosocial object^(12)^.

Since all reactors were eligible, it was decided that those that could not be located and those that, although interested, expressed inability to schedule an interview after three attempts would be excluded, with 27 (36.49%) being excluded, with no refusals or withdrawals. The others (n = 10; 13.51%) were not contacted, as the sampling recommendation and data saturation were met.

### Data Collection

Initially, the research project was presented to the course coordinator for institutional authorization and the provision of a room to conduct the interviews. With prior authorization granted by the students, the team of researchers involved in the testing provided the first author with a list of names, contacts, and specific information on the reactors testing. Thus, they were contacted by the first author, via instant messaging application or telephone call, at which time he provided some information about the research to invite them to participate.

With those who accepted, a meeting was scheduled to take place in the reserved room, with the aim of detailing and clarifying the objectives, procedures, risks and benefits, obtaining formal/voluntary acceptance and carrying out an individual interview, a production technique that is in line with the procedural aspect of the TSR^(9)^. Since the first author was part of the testing team and carried out activities to allow the application, reading, and interpretation of the tuberculin test, the students met him beforehand and were informed about his academic/professional trajectory and aspirations, including the fact that this study was a mandatory requirement to complete the master’s degree in nursing.

From March to July 2023, the interviews were conducted by the first author, trained in guidance meetings, as curricular activities of the postgraduate course, and in regular meetings of a research group linked to the authors’ institution. To ensure comfort and privacy, in compliance with some health recommendations to control the COVID-19 pandemic, still in force at that time, only the participant and the first author occupied the room, which was kept ventilated, 70% alcohol gel was provided, and the participant was informed that he/she could use a mask, although this procedure was no longer mandatory at the time the data were produced. The interviews lasted a minimum of 20 and a maximum of 50 minutes, and were audio-recorded in MP3 format using an electronic device.

To guide them, a semi-structured script was created, consisting of two axes. With questions to know the sociodemographic and academic profile of participants, demonstrating their social belonging^(9)^, the first axis investigated the variables age, sex, grade, and semester of the course, color/race, religion, marital status, number of children, receipt of scholarship and funding institution, occupation, monthly family income, municipality of origin, number of people with whom they lived and who these people were, in addition to the size of the induration generated by the tuberculin test. With subjective questions, the second axis investigated knowledge, opinions, and experiences on the topic to understand the SR.

This instrument was not subjected to pilot testing, but was evaluated by four PhD professors, linked to Postgraduate Studies Programs in Nursing, selected for having notable intellectual production and researching topics related to higher education, nursing, and public health. Among them, two endorsed the instrument and two made suggestions, the pertinent aspects of which were accepted for its qualification. Given the completeness of the data, it was not necessary to repeat interviews or incorporate other production techniques. Not to compromise the spontaneity of the statements, the transcripts were not shared with the participants.

### Data Analysis and Treatment

The sociodemographic and academic profile data were tabulated in a *Microsoft Office Excel*® spreadsheet (version 2013) and analyzed with descriptive statistics to highlight the absolute and relative numbers, underscoring the predominant results, except in relation to the variables grade/semester period, marital status, and municipality of origin, detailed in full. The subjective data were transcribed to form a text *corpus* in a single file, without formatting (.txt file type), imported into the open access software *Interface de R pour les Analyses Multidimensionnelles de Textes et de Questionnaires* (IRaMuTeQ®, version 0.7, alpha 2), to perform lexical analysis. By using functions of the software R, the IRaMuTeQ® generates statistical and semantic data from the processing of various sources that deal with a single theme, such as a set of transcribed interviews, helping to identify their meanings^(13)^.

Five analytical modalities are offered in its menu: simple statistics (lexicographic analysis), specificities and correspondence factor analysis, descending hierarchical classification (DHC), similarity analysis, and word cloud. Among them, DHC was chosen, with the aim of forming lexical classes that express the content of the *corpus* and are illustrated by a horizontal dendrogram, which points out the partition logic of the *corpus* and the percentages of text segments (TS) that constitute the classes. The words associated with them are characterized by four statistical values: frequency of TS containing the word in the class (Fc); total frequency of TS containing the word in the *corpus* (Ft); percentage (%) of TS that contains the word in the class, in relation to its occurrence in the *corpus*; and chi-square (X^2^), which demonstrates the associative strength of the word, considering it representative when p < 0.0001^(13)^.

Data were interpreted in light of the scientific literature and the cognitive processes that form SR, which are based on social memory and past conclusions, and are known as anchoring and objectification. The first consists of familiarizing an object, initially seen as strange and distinct from reality, allowing its classification and (re)naming, according to the group’s precepts and values. The second consists of the materialization of the object through an image or symbol that visually characterizes it, reasons why SR depends on individual and collective experiences, with every representation being constituted by a dual nature, conceptual and figurative, pointing to these processes^(8)^.

### Ethical Aspects

The guidelines of Resolution No. 466/2012 of the National Health Council/Ministry of Health were followed, obtaining institutional authorization and approval by the Research Ethics Committee of the Undergraduate Nursing Course at the university where the study took place, with Opinion No. 5.458.024, issued in June 2022. All participants read and signed the Free and Informed Consent Form, declaring their acceptance, prior to the interviews. To ensure the confidentiality of their identities, an alphanumeric code was used, consisting of the letter E, for “student” in Portuguese (“*estudante*”), followed by a Roman numeral (which indicates the course grade), a hyphen and a cardinal number (which indicates the order of the interviews).

## RESULTS

### Sociodemographic and Academic Profile of Participants

Age ranged between 19 and 40 years, with the age groups 19 to 23 (n = 28; 75.68%) and 24 to 28 years (n = 6; 16.22%) standing out, with 24 (64.86%) being women. Regarding the grades and semester periods, there were: one (2.70%) in the 1st grade/block I and six (16.22%) in the 1st grade/block II, totaling seven enrolled (18.92%) in the 1st grade; two (5.41%) were in the 2nd grade/block I and three (8.11%) were in the 2nd grade/block II, totaling five enrolled (13.51%) in the 2nd grade; eight (21.62%) were in the 3rd grade/block II; four (10.81%) were in the 4th grade/block I and six (16.22%) were in the 4th grade/block II, totaling 10 enrolled (27.03%) in the 4th grade; four (10.81%) were in the 5th grade/block I and three (8.11%) were in the 5th grade/block II, totaling seven enrolled (18.92%) in 5th grade.

Thus, the 3rd grade/block I was the only period in which there were no participants, due to the impossibility of agreeing on days and times suitable for the interviews. However, this did not constitute a limitation, as the other periods were covered, characterizing the effective participation of all grades.

Regarding color/race, 21 (56.76%) declared themselves to be brown and 11 (29.73%) white. Regarding religion, 17 (45.95%) were Evangelicals and 14 (37.84%) were Catholic. Regarding marital status, 32 (86.49%) were single, four (10.81%) lived in a consensual union, and one (2.70%) was married, with the majority (n = 35; 94.59%) stating that they had no children. It was found that 22 (59.46%) did not receive a scholarship funding and, among those who did, 14 scholarships came from public education, assistance or health management institutions, mainly for extracurricular internships (n = 7; 18.92%).

Furthermore, 31 (83.78%) reported not having professional activities, 23 (62.16%) had a monthly family income between two and three minimum wages, and eight (21.62%) had above five, considering that, in Brazil, in 2023, the official salary value was R$ 1,320.00. Regarding the municipality of origin, 28 (75.68%) lived in Belém and nine (24.32%) in other municipalities in the Metropolitan Region. The number of people they lived with varied between one and 18, with three people prevailing (n = 13; 35.14%), mainly mother, siblings and father, mentioned by 25 (67.57%), 18 (48.65%), and 13 (35.14%) participants, respectively. Regarding the tuberculin test, it was found that the size of the induration varied between five and 20 mm, with a predominance of 10 (n = 13; 35.14%) and 15 (n = 7; 18.92%), with an approximate average of 11.70 mm.

### Presentation of the Results of the Text Corpus

The IRaMuTeQ® identified 37 texts, corresponding to the set of interviews. Through DHC, the texts were broken down into 1,939 TS, resulting in the use of 1,686 (86.95%), with 67,619 occurrences (forms or words) identified, of which 5,022 were distinct words and 2,367 were hapaxes (words with a frequency equal to one), corresponding to 47.13% of the distinct words and 3.50% of the occurrences. The average number of occurrences per TS was 34.87.

**Figure 1:**
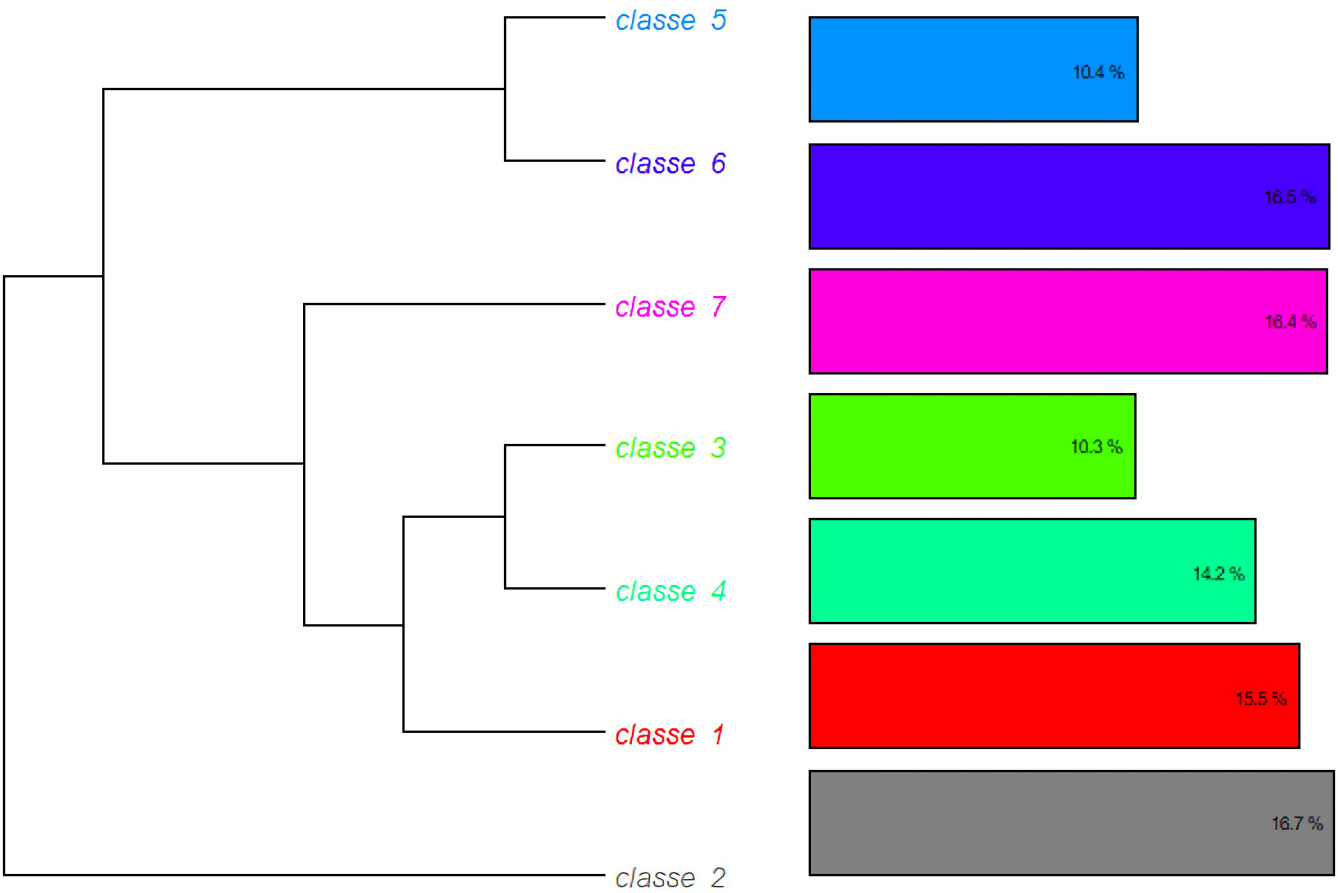
Horizontal dendrogram of descending hierarchical classification – Belém, Pará, Brazil, 2023.

Note: *classe* = class.

Through the partition logic of the *corpus*, seven lexical classes were generated, forming two *subcorpora*, whose classes presented different absolute and relative numbers of TS. Thus, the first *subcorpus* was constituted by class 2 (with 281 TS; 16.67% of the *corpus*), and the second, respectively by class 5 (175 ST; 10.38%), class 6 (278 ST; 16.49%), class 7 (277 ST; 16.43%), class 1 (262 ST; 15.54%), class 3 (174 ST; 10.32%), and class 4 (239 ST; 14.18%), as evidenced by the horizontal dendrogram of the DHC, whose percentages were rounded, by IRaMuTeQ®, with one decimal place after the decimal point (Figure 1).

To meet the objective of this study, class 7 was chosen to be detailed, which emerged from the second *subcorpus* and presented 65 representative words (p < 0.0001), among which 21 (32.31%) stood out, especially for their chi-square, as shown in Table 1.

**Table 1:** Details of the most representative words in class 7 – Belém, Pará, Brazil, 2023.

No.	Words	Fc	Ft	%	X^2^
1	Information	121	175	69.14	395.18
2	Course	94	123	76.42	347.80
3	Lack	51	58	87.93	223.66
4	Subject	57	84	67.86	170.29
5	To_study	40	50	80.00	151.66
6	Curricular_component	30	34	88.24	130.31
7	Curricular_components	23	24	95.83	111.80
8	IPD	36	52	69.23	108.95
9	Nursing_students	24	27	88.89	104.93
10	Semester	18	19	94.74	85.82
11	Beginning	19	22	86.36	79.40
12	Microbiology	13	13	100.00	66.64
13	To_obtain	15	17	88.24	64.49
14	Knowledge	36	72	50.00	61.73
15	To_minister	12	12	100.00	61.48
16	Eighth_semester	12	13	92.31	54.94
17	Before	43	101	42.57	53.49
18	Student	26	47	55.32	53.26
19	To_address	10	10	100.00	51.17
20	In_depth	12	14	85.71	49.36
21	To_learn	17	26	65.38	46.09

Note: IPD: infectious and parasitic diseases; Fc: frequency of TS containing the word in the class; Ft: total frequency of TS containing the word in the *corpus*; %: percentage of TS that contains the word in the class, in relation to its occurrence in the *corpus*; X^2^: chi-square.

According to its content, this class was named by the authors as “Informing about LTBI” and organized into a thematic axis, called “Informing to better care: challenges and possibilities in higher education”, aiming to present the subjective data, which outlined two representational sets.

The way participants perceived the conditions in which information about LTBI was shared during the nursing course is addressed, constituting the first representational set. In this sense, they pointed out suggestions that, in their view, could contribute to solving the challenges inherent to this context, or mitigate its harmful effects on the personal, academic, and professional daily lives of students, constituting the second representational set, as detailed below and exemplified with some emblematic excerpts.

### Thematic Axis – Informing to Better Care: Challenges and Possibilities in Higher Education

The TS in class 7 revealed that the undergraduate course they experienced lacked qualified and timely information about LTBI, which was not only presented in isolation, but reiterated and better grounded in everyday academic life. This understanding was anchored by two coexisting factors, which limited the global understanding of the topic, considering that the students represented information as an essential element for dealing with LTBI.

The first factor corresponded to the premise that, often, the topic was introduced late, during the curricular component Nursing in Infectious and Parasitic Diseases (IPD), in the eighth semester of the course. In turn, the second was related to the fact that, regardless of the school period in which the topic was taught, its presentation often occurred at a specific time, with superficial content by professors, apparently not giving it due importance.

The two most representative words, “information” (X^2^ = 395.18) and “course” (X^2^ = 347.80), associated with words like “lack” (X^2^ = 223.66), “subject” (X^2^ = 170.29), “IPD” (X^2^ = 108.95), “semester” (X^2^ = 85.82), “microbiology” (X^2^ = 66.64) and “in-depth” (X^2^ = 49.36), ratify these factors, strongly anchored in the idea that they configure a negative reality, which is why they were objectified by terms that refer to the superficiality of content, such as “very superficial” and “high school style”, expressed by EIII-7:

*I think it lacks depth, as we see, for example, in Microbiology and Pathology, but I found it very superficial, very high school style. So much so that, if you ask me something more in-depth about the subject, I won’t know how to answer and, personally, I find that embarrassing. This is both my fault, for not having gone into more depth, and the fault of the teaching in the course. What we had was a class* [in Microbiology] *about this, along with several other subjects, in an entire morning, and a handout from a very old book.* (EIII-7)

*I think there is lack of information, because, for example, I only found out that this test existed* [tuberculin test] *because I did it, but I had no idea that this condition, LTBI, existed. More visibility is required, because we study TB, we have classes about the microorganism, but we don’t usually talk about LTBI, which can be in the latent form.* (EII-19)

*Before the research* [referring to testing with the tuberculin skin test], *I had already come into contact with a TB patient, but I had no concrete idea about LTBI. I knew what TB was, how treatment was carried out, how to fill out the notification form, and I knew about some tests, but I only found out the details six months after coming into contact with this patient* [learned details in the curricular component Nursing in IPD]. (EIV-20)

*There is lack of information about this among students, because at that time* [when he/she received the test result], *I didn’t understand. I think I still have doubts that I need to study and talk to professionals about, so as not to reinforce the stigma that exists about the disease and LTBI.* (EIV-29)

To overcome the challenges posed by the lack of information, or mitigate its effects, the participants made suggestions that address two main aspects: 1) the need to rethink and restructure the curricular matrix so that the topic is taught prior to the eighth semester, in the curricular component Nursing in IPD and/or in other component(s), with pertinent teaching-learning strategies, anchoring themselves in the understanding that the eighth semester is too late to introduce the topic and that uninteresting strategies can hinder the sharing of information; 2) to promote different spaces for dialogue and training in the university environment, objectified in the formats of lectures and complementary courses, to clarify doubts and enhance the dissemination of the topic, considering its relevance.

These suggestions are corroborated by the occurrence of the verbs “to study” (X^2^ = 151.66), “to minister” (X^2^ = 61.48), “to address” (X^2^ = 51.17) and “to learn” (X^2^ = 46.09), together with the words that indicate temporality, “beginning” (X^2^ = 79.40) and “before” (X^2^ = 53.49). The first suggestion was expressed, for example, in the statements by EIV-29 and EIII-34, and the second, in those by EIII-7 and EIII-25:

*I think that LTBI is a condition that we study a lot at the end of the course, and we could start discussing it earlier, for example, in Anthropology, where we study our relationship with others. The university could provide examples within curricular components such as Anthropology and Philosophy, in the context of sick people, so that we can reinforce the view of the sick person and the transmission of the disease in a more ethical and respectful way and, thus, stimulate learning.* (EIV-29)

*Talking to people who participated in the test, I realized that many of them were not familiar with LTBI, they didn’t know it existed. In the course curriculum, there should be a curricular component to address this subject earlier, in the first semesters, as I don’t know if I’ll still study. But it should have, because we end up seeing much further ahead, when we have already been through several scenarios where there were people with TB, and this situation is complicated.* (EIII-34)

*This could be solved with more courses on the subject. For example, I went to a lecture about TB at a certain hospital, and it was very good, I learned a lot of things that I didn’t learn at school and university. I learned that there is not only pulmonary TB, as the doctor who gave the lecture spoke about the disease affecting several other organs.* (EIII-7)

*I think this can be resolved with lectures and exchange of information, not necessarily in a specific semester, but from the beginning of the course, such as in the Microbiology component.* (EIII-25)

## DISCUSSION

As a first representational set, the results present a delicate and concerning reality, which is why it needs to be reviewed by the competent academic-administrative agencies. According to the participants, this reality was configured by the fact that the course presented limitations regarding the timely sharing of information about LTBI throughout the academic periods, culminating in weaknesses in academic and professional training.

It is known that the practices of individuals and groups, such as actions of care for oneself and others, symbolize the realization of SR, since this form of particular knowledge guides communications and behaviors among people^(8)^. However, it is necessary that information about a given object of subjective importance be conveyed, as it is an important element in the elaboration of the SR (information dimension), as defended by other authors who carried out studies in interface with the TSR^(9,14)^.

Thus, it is possible to infer that, without adequate information, shared at an opportune time and reiterated at other points in the course, students may not feel capable or confident to effectively carry out their self-care and care practices aimed at human groups, such as users of health services in which they carry out supervised activities.

In dialogue with this evidence, it should be highlighted that, in the context of academic training, a qualitative study, carried out with 25 students in the third semester of the Undergraduate Nursing Course at a public university in the South of Brazil, analyzed the contributions of skills training through simulation, in a course discipline, as a pedagogical strategy to develop skills. Among other results, the training allowed the strengthening of the skills and safety to perform technical procedures, prior to classes in real clinical scenarios, based on theoretical content and feedback from the professor regarding the execution of the procedures^(15)^.

This experience provided an opportunity to better manage feelings such as anxiety, insecurity, and nervousness, which manifest themselves when faced with the challenges of training. Furthermore, it reiterated knowledge, clarified doubts, and established attitudes of self-assessment and intellectual autonomy, mobilizing students to organize themselves and become co- responsible for their learning. Thus, with the professor’s teachings, they recognized the need to study to deepen their theoretical and practical knowledge of the discipline, directly influencing the quality of care actions^(15)^.

Justifying the limitations of information on LTBI, participants anchored them in two factors. The first factor corresponded to the late introduction of the topic, in the eighth semester of the course, and the second showed that, despite its importance, the topic was approached in an isolated and superficial manner, leading students to not understand it adequately and not relate it to other content and/or curricular components, weakening the teaching-learning process.

In higher education, the training of human resources for the health field requires that, to be effective, this process be critical-reflective and endowed with systematic experiences, especially in the reality of the SUS. Such experiences should enable the internalization of content and the creation of mental networks that associate them in a coherent manner, with the aim of generating meanings that guide professional practices^(16)^, a context that signals and reinforces the pragmatic nature of RS.

When it comes to human health, it is necessary to consider that the individual, the family, and the community are the target subjects of these practices, which is justified by the fact that they are complex, multi-determined, and not compartmentalized. Thus, they express biopsychosocial characteristics, which must be valued so that their individual and collective needs are adequately met^(17)^.

This context points to the five work processes in nursing (assistance, administration, teaching, research, and political participation) as aspects that materialize the scientific, ethical, political, and social responsibility of the profession^(18)^. Such processes must be assumed by nurses in their daily work, depending on the nature of the activities they perform with human groups. For this reason, higher education must enable them to deal with the challenges and possibilities of the profession.

To address the limitations regarding sharing the theme, the students revealed the second representational set, inherent to the suggestions that, in their view, if implemented, they could solve the limitations or mitigate their effects. These suggestions were divided into two aspects, which represent, in the first instance, the need to rethink/restructure the course’s curricular matrix, anchored in the idea of sharing the topic at an opportune moment, and in the second instance, to encourage training spaces to discuss the topic, objectivated in different formats.

Thus, the idea of joining efforts (training spaces) together with mandatory curricular activities (provided for in the curricular matrix) became clear, to clarify doubts and strengthen the sharing of the topic with students, in different school periods, considering that they could be exposed to the bacillus, during practical activities, from the beginning of the course. This representation is supported by the literature, due to the frequent contact of these actors with the community and with institutional settings in public health and hospital care^(5,6,7)^.

Therefore, in light of the results, it can be inferred that the possibility of exposure was represented by students as a factor that justifies the need to be introduced to the topic prior to the eighth semester and to discuss it throughout the course, to have access to qualified information that helps them develop self-care, through biosafety practices to prevent LTBI in the community and in health services, and to develop better care practices aimed at human groups.

In view of this, it is appropriate to consider that the reality experienced by students antagonizes the expanded concept of health, collectively constructed at the VIII National Health Conference, held in 1986, and legitimized in the Federal Constitution of 1988, a context in which health became a right of all citizens and a duty of the State. Based on this concept, Law No. 8.080/1990, which regulated the SUS, declares that health is multidetermined, as it results from environmental, educational, housing, nutritional, occupational, leisure, freedom, access to land ownership, and access to health services, among others^(19)^.

This expanded conception comes from a historical process, which involves human relations and man’s ability to reflect on his reality^(20)^, constituting not a mere academic whim, but a political necessity^(21)^. This allows us to state that, without quality information and educational processes, the well-being of the individual and of his community can be weakened, since information, knowledge, and communication are associated with the promotion of health and the prevention of diseases^(22)^, establishing relationships with the expanded concept of health^(17,19)^.

The students implied the need for professors to develop differentiated teaching strategies in their curricular components, to stimulate learning about LTBI. It is understood that this points to the appropriate use of active learning methodologies, and that the academic community must continually reflect on the importance of these methodologies, apply them and encourage them, considering their potential to train qualified human resources. Thus, in the health field, including nursing education, these methodologies stand out in the national^(23,24)^ and international^(25,26)^ literature.

From this perspective, when suggesting that the competent authorities promote training spaces on LTBI, objectivated through courses and lectures in the university environment, the students made their desire to be actively involved in these spaces clear, participating and contributing to their implementation, as evidenced by the expression “exchange of information”, which points to a dialogical process.

Based on the SRs revealed here and the connections they maintain among themselves, it is understood that the limitations of information about LTBI, in undergraduate nursing courses, constitute a driving force that hinders, at least partially, the consolidation of the teaching-learning process, with possible negative repercussions on the training of nurses, depending on the degree of limitations.

Reiterating the importance of information for actions in the context of TB, methodological research with a qualitative approach, carried out with 41 nurses who worked in PHC units in the city of Belém, revealed the lack of knowledge about standards and guidelines of the TB Control Program among the difficulties, reported by them, to control cases of the disease. These standards and guidelines were related to case management, the indication of laboratory tests, and the need to refer the patient to secondary or tertiary care, weakening care and epidemiological surveillance actions^(27)^.

To address this scenario, they suggested that an educational technology, in documentary format, be developed and made available in the daily lives of nurses working on TB control. In this respect, they pointed out the need to contain information that helps professionals to develop their activities in PHC, including specific information about LTBI, as it is understood as a health condition with different characteristics compared to TB. This research resulted in the participatory construction of an instructive guide, aiming to meet the local and regional peculiarities of the scenario in which it was carried out^(27)^.

Identifying similarities with another topic, it is clear that the lack of information does not only occur with LTBI. As an example, an integrative review is cited, with 10 national and international primary studies, which analyzed evidence on nursing care for children with special health needs in PHC. Among other results, it was demonstrated that most nurses do not feel qualified to care for this population, due to limitations in their academic training and/or regular and specific professional training^(28)^.

It is also worth considering that the limitations of information conflict with the current National Curricular Guidelines (*Diretrizes Curriculares Nacionais* – DCN) for the Nursing Undergraduate Course, established by Resolution No. 3, of November 7, 2001, of the National Education Council^(29)^. This reflection is based on the fact that these limitations constitute a reality contrary to what the DCN guides, that is, the training of professionals with technical-scientific, ethical and humanistic skills and abilities, aware of their social responsibility, to meet individual and collective health needs, aiming to mobilize transformations in the reality of human groups.

Nevertheless, it is also necessary to consider the student’s co-responsibility for his/her education, given that he/she must make every possible effort to resolve his/her knowledge weaknesses and to strengthen his/her skills and abilities. To this end, higher education must encourage critical-reflective attitudes, through emancipatory processes, which generate intellectual autonomy and, thus, mobilize the active search for knowledge^(23)^.

Furthermore, the teaching-learning process must be constructed collectively by professors and students, which is why the active participation of both is fundamental and non-transferable^(25)^. In this sense, relationships between students are equally important for establishing collaborative networks that help them overcome the challenges of the teaching-learning process^(30)^, such as weaknesses in the understanding of certain topics, such as LTBI, and their coherent application in self-care practices and in care practices aimed at human groups.

As a limitation of this study, it is worth mentioning that it was carried out in the Undergraduate Nursing Course at a public university in Belém, configuring a specific scenario, which is influenced by several conditions, such as cultural, economic, political, social and territorial factors. This characterizes a small geographic representation of the data, considering that this municipality has other Undergraduate Nursing Courses, mainly linked to private HEIs, which were not considered due to the restrictions imposed by the COVID-19 pandemic. However, it is understood that the SRs revealed here can, at least partially, converge with the reality experienced/faced by nursing students from these and other HEIs in regional and national scenarios.

Thus, the results can help students, professors, and other actors in the academic community to review/restructure curricular matrices and guide teaching-learning processes in higher education, aiming to improve the sharing of information about LTBI and qualify academic and professional training. Furthermore, they can contribute to the competent university bodies and public authorities to reflect on the need to strengthen, from the perspective of intersectorality between the fields of health and education, biosafety conditions in health services, with the aim of preventing cases of LTBI in this public, also strengthening control and epidemiological surveillance actions related to this problem.

## CONCLUSION

This study demonstrated that nursing students’ SR regarding information sharing on LTBI outlined two representational sets inherent to their experiences with the topic in higher education. These sets pointed out limitations that weakened the timely sharing and quality of information, in addition to suggestions that, in the students’ view, if implemented, could resolve limitations or mitigate their effects.

Given these results, it is worth highlighting that, in addition to its contributions to nursing education, academic management in higher education, and the management of health services in which students develop practical activities, this study can contribute to a better understanding of intersectoral objects or phenomena involving health and education, such as the psychosocial object investigated here.

In this sense, the results have the potential to support the theoretical-methodological conception of research that aims to explore other aspects related to LTBI among nursing students, valuing the qualitative approach, especially in its interface with TSR, considering the potential of this theory to reveal, systematize, and interpret subjectivities in the daily lives of individuals and groups, which is why it has been heavily used by health and education researchers.

## Data Availability

The data supporting this study are available upon request to the corresponding author.
