# Supplementary material for: Information on latent tuberculosis infection in higher education: social representations of nursing students
Source: Rev Esc Enferm USP. 2025 Sep 8;59:e20250029. doi: 10.1590/1980-220X-REEUSP-2025-0029en (PMC12448237; doi:10.1590/1980-220X-REEUSP-2025-0029en)
Supplement: Translation

# Translation: Informações sobre a infecção latente da tuberculose na educação superior: representações sociais de estudantes de enfermagem*

**DOI:** 10.1590/1980-220X-REEUSP-2025-0029pt

**Authors:** Erlon Gabriel Rego de Andrade, Ivaneide Leal Ataíde Rodrigues, Laura Maria Vidal Nogueira, Eliza Paixão da Silva, Adriely Alciany Miranda dos Santos, Letícia Gabriela Noronha Rodrigues, Débora de Cássia Quaresma Silva, Vitória de Cássia Quaresma Silva

**Affiliations:** 1 Universidade do Estado do Pará, Escola de Enfermagem Magalhães Barata, Programa de Pós-Graduação em Enfermagem, Belém, PA, Brasil.; 2 Universidade do Estado do Pará, Centro de Ciências Biológicas e da Saúde, Escola de Enfermagem Magalhães Barata, Belém, PA, Brasil.; 3 Universidade Federal do Pará, Instituto de Ciências da Saúde, Faculdade de Enfermagem, Belém, PA, Brasil.

**Keywords:** Tuberculose Latente, Estudantes de Enfermagem, Universidades, Representação Social, Psicologia Social

## Abstract

**Objetivo:**

Analisar as representações sociais de estudantes de enfermagem acerca do compartilhamento de informações sobre a infecção latente da tuberculose na educação superior.

**Método:**

Estudo descritivo, qualitativo, ancorado na vertente processual da Teoria das Representações Sociais. Foi realizado com 37 estudantes reatores à prova tuberculínica, matriculados da 1ª à 5ª série, no Curso de Graduação em Enfermagem de uma universidade pública em Belém, Pará, Brasil. Entre março e julho de 2023, realizaram-se entrevistas individuais semiestruturadas, cujo *corpus* foi submetido à análise lexical com o *software Interface de R pour les Analyses Multidimensionnelles de Textes et de Questionnaires* (0.7, *alpha* 2), utilizando a classificação hierárquica descendente.

**Resultados:**

Identificaram-se 1.939 segmentos de texto, dos quais foram aproveitados 1.686 (86,95%), gerando sete classes lexicais. Optou-se por detalhar a classe 7, que delineou dois conjuntos representacionais, abordando como as informações eram compartilhadas e sugestões sobre o tema.

**Conclusão:**

As limitações que fragilizavam o compartilhamento oportuno e a qualidade das informações motivaram os estudantes a tecer sugestões para solucionar os desafios inerentes a esse contexto ou mitigar seus efeitos deletérios.

## INTRODUÇÃO

Na atualidade, a tuberculose (TB) ainda representa importante problema de saúde pública, devido à ocorrência das formas pulmonar e extrapulmonar, respectivamente caracterizadas pelo acometimento dos pulmões e de outras estruturas anatomofisiológicas, marcadas pela multiplicação e propagação do *Mycobacterium tuberculosis*. Outra preocupação consiste no estado que antecede o adoecimento, conhecido como infecção latente da tuberculose (ILTB), que resulta da exposição ao bacilo expelido por pessoas com TB pulmonar ou TB laríngea sem tratamento e caracteriza-se por sua instalação nos pulmões, gerando resposta imunológica adaptativa, sem desencadear sinais e sintomas, não havendo possibilidade de transmissão^(1,2)^.

Vivendo com o bacilo, pessoas imunocompetentes podem nunca manifestar a TB, mas evidências demonstram que o maior risco de adoecimento ocorre nos dois primeiros anos após a infecção, sobretudo entre grupos com vulnerabilidade imunológica, como aqueles com idade abaixo de dois ou acima de 60 anos, em uso de terapia imunossupressora, que vivem com o vírus da imunodeficiência humana (HIV), com diabetes *mellitus* e/ou outras doenças crônicas não transmissíveis^(2)^. Estimativas apontam que a ILTB acomete cerca de um quarto da população global, configurando grande contingente de suscetíveis^(3)^.

Fornecida pelo Sistema Único de Saúde (SUS), a prova tuberculínica é o teste predominante para diagnosticar a ILTB no Brasil. Requer profissional habilitado para aplicar, por via intradérmica, 0,1 ml de tuberculina no terço médio da face anterior do antebraço, gerando reação de hipersensibilidade cutânea, cuja enduração deve ser medida, considerando o maior diâmetro transverso. Utiliza-se régua milimetrada para ler e interpretar o teste entre 48 e 72, ou até 96 horas, após a aplicação, devendo-se registrar o resultado em milímetros (mm), inclusive quando não houver enduração (zero mm), considerando reator (infectado) o indivíduo com enduração igual ou superior a cinco milímetros^(4)^.

Para desenvolver estratégias de controle efetivas, alinhadas com a vigilância epidemiológica, é necessário investigar a ILTB, sobretudo nos grupos que se caracterizam por maior exposição ao bacilo, como estudantes das áreas da saúde, entre os quais destacam-se os estudantes de enfermagem, pois estudos já revelaram alta prevalência de infecção nesse público^(5,6)^. Tal exposição ocorre porque, durante o curso, esses estudantes são inseridos em atividades práticas supervisionadas, como as que são realizadas na Atenção Primária à Saúde (APS), por meio dos programas de saúde coletiva, com diferentes grupos humanos, destacando-se as pessoas com TB e seus acompanhantes, cuidadores e familiares^(7)^.

Além de os aspectos clínico-epidemiológicos serem relevantes para compreender o tema, entende-se que aspectos psicossociais atravessam as experiências dos estudantes frente às situações de exposição e ao possível contágio, pois, uma vez infectados e cientes da sua condição a partir do diagnóstico, os estudantes podem alterar a maneira como compreendem a sua realidade e as relações nela tecidas com outros atores, a exemplo dos usuários dos serviços de saúde, internalizando a necessidade de empreender ou transformar práticas de autocuidado, para evitar o adoecimento, e práticas de cuidado direcionadas aos grupos humanos, para atender às suas necessidades biopsicossociais. Assim, na educação superior, o acesso oportuno a informações de qualidade sobre a ILTB, durante a formação universitária, pode favorecer tais práticas.

Isso aponta para a Teoria das Representações Sociais (TRS), originária da psicologia social e formulada por Serge Moscovici, na década de 1960. Visa compreender as representações sociais (RS), uma forma de conhecimento que guia estratégias com as quais os indivíduos se comunicam, interagem e se comportam em sociedade^(8)^. As RS estabelecem uma linguagem comum, que particulariza os grupos frente às suas pertenças sociais, isto é, características que apontam diferenças nas condições de produção das RS^(9)^.

Exibem três dimensões basilares: a informação, definida pelos saberes sobre o objeto psicossocial e adquirida por meio das experiências; a atitude, marcada pelo julgamento que o indivíduo faz acerca do objeto, induzindo-o a um posicionamento simbólico; e campo de representação ou dimensão da imagem, que idealiza um modelo social, com elementos representacionais dispostos de maneira ordenada e hierarquizada. Portanto, as informações tem papel crucial na produção das RS e ajudam a compreender as práticas cotidianas^(8)^.

Frente à relevância do tema, elaborou-se a questão norteadora: como estudantes de enfermagem representam o compartilhamento de informações sobre a ILTB na educação superior? Para respondê-la, este estudo objetivou analisar as representações sociais de estudantes de enfermagem acerca do compartilhamento de informações sobre a infecção latente da tuberculose na educação superior.

## MÉTODO

### Tipo de Estudo

Estudo descritivo, com abordagem qualitativa, ancorado na vertente processual da TRS e guiado pelo instrumento *Consolidated Criteria for Reporting Qualitative Research* (COREQ), considerando seus 32 itens, distribuídos em três domínios, inerentes à equipe de pesquisa, ao desenho do estudo e à análise e apresentação dos resultados^(10)^. Coadunando com essa abordagem, a vertente processual da TRS possibilita compreender o processo de construção das RS, revelando seus elementos constitutivos, que podem ser identificados, por exemplo, mediante a expressão de aspectos cognitivos, ideológicos, imagéticos e informativos, ou que denotam atitudes, crenças, opiniões e valores sociais^(9)^.

### Local

Ocorreu no Curso de Graduação em Enfermagem de uma universidade pública no município de Belém, estado do Pará, Brasil. Com duração de cinco anos, o curso é operacionalizado em cinco séries e 10 períodos semestrais (cada série constituída por dois blocos, identificados como bloco I e bloco II), totalizando 5.000 horas de atividades teóricas e práticas^(11)^. Esse cenário foi escolhido por suas características atenderem ao interesse de investigar o tema no cotidiano de estudantes de enfermagem.

### População e Critérios de Seleção

Participaram 37 estudantes reatores à prova tuberculínica, com resultado igual ou superior a cinco milímetros, regularmente matriculados e cursando da 1ª à 5ª série. Os estudantes foram previamente testados como parte das atividades de uma pesquisa multicêntrica, desenvolvida no âmbito do Programa Nacional de Cooperação Acadêmica (PROCAD), ofertado pela Coordenação de Aperfeiçoamento de Pessoal de Nível Superior (CAPES/Ministério da Educação) para estimular redes colaborativas entre Programas de Pós-Graduação *Stricto Sensu*, incluindo Programas de Pós-Graduação em Enfermagem. Essa pesquisa envolveu quatro instituições de ensino superior (IES) públicas, localizadas nas capitais de quatro estados brasileiros: Belém (Pará), Manaus (Amazonas), Rio de Janeiro (Rio de Janeiro) e Terezinha (Piauí).

Em Belém, a testagem ocorreu entre dezembro de 2022 e fevereiro de 2023, período no qual havia, aproximadamente, 345 estudantes matriculados no curso. Foram testados 269 (77,97% de aderência), identificando-se 74 reatores (27,51% dos testados), sendo incluídos 37 (50,00%) neste estudo. Esse número atendeu à recomendação amostral de 20 a 30 participantes, apontada na literatura como empiricamente satisfatória para alcançar objetivos em pesquisas qualitativas, e considerou a saturação dos dados, evidenciada quando estes se mostraram suficientes e, portanto, acréscimos não modificariam a compreensão dos autores acerca do objeto psicossocial^(12)^.

Visto que todos os reatores eram elegíveis, optou-se por excluir os que não fossem localizados e os que, embora interessados, manifestassem impossibilidade de agendamento para realizar entrevista após três tentativas, sendo excluídos 27 (36,49%), não havendo recusas ou desistências. Os demais (n = 10; 13,51%) não foram contactados, já que a recomendação amostral e a saturação dos dados foram atendidas.

### Coleta dos Dados

Inicialmente, apresentou-se o projeto de pesquisa à coordenadora do curso para solicitar a autorização institucional e a cessão de uma sala para realizar as entrevistas. Mediante autorização prévia, concedida pelos estudantes, a equipe de pesquisadores envolvidos na testagem disponibilizou, ao primeiro autor, uma lista com nomes, contatos e informações específicas da testagem dos reatores. Assim, foram contactados pelo primeiro autor, via aplicativo de mensagens instantâneas ou ligação telefônica, oportunidade na qual forneceu algumas informações sobre a pesquisa para convidá-los a participar.

Com os que aceitaram, agendou-se um encontro para ocorrer na sala reservada, visando pormenorizar e esclarecer os objetivos, procedimentos, riscos e benefícios, obter o aceite formal/voluntário e realizar entrevista individual, técnica de produção que coaduna com a vertente processual da TRS^(9)^. Visto que o primeiro autor compunha a equipe de testagem e operacionalizou atividades para possibilitar a aplicação, leitura e interpretação da prova tuberculínica, os estudantes o conheceram previamente e foram informados sobre sua trajetória e aspirações acadêmicas/profissionais, incluindo o fato de que este estudo figurava como requisito obrigatório para concluir o curso de mestrado em enfermagem.

No período de março a julho de 2023, as entrevistas foram conduzidas pelo primeiro autor, capacitado em reuniões de orientação, que compuseram as atividades curriculares da pós- graduação *stricto sensu*, e em encontros regulares de um grupo de pesquisa vinculado à instituição dos autores. Para garantir conforto e privacidade, atendendo a algumas recomendações sanitárias para controlar a pandemia de COVID-19, ainda vigentes naquele momento, apenas o participante e o primeiro autor ocuparam a sala, mantendo-a arejada e dispondo de álcool em gel 70%, informando-se a possibilidade de utilizar máscara, embora esse procedimento já não fosse obrigatório no período em que os dados foram produzidos. As entrevistas tiveram duração mínima de 20 e máxima de 50 minutos, sendo audiogravadas no formato MP3, utilizando dispositivo eletrônico.

Para norteá-las, elaborou-se um roteiro semiestruturado, constituído por dois eixos. Com perguntas para conhecer o perfil sociodemográfico e acadêmico dos participantes, demonstrando sua pertença social^(9)^, o primeiro eixo investigou as variáveis idade, sexo, série e período semestral do curso, cor/raça, religião, situação conjugal, número de filhos, recebimento de bolsa de estudos e instituição de fomento, ocupação, renda familiar mensal, município de origem, número de pessoas com quem residiam e quem eram essas pessoas, além do tamanho da enduração gerada pela prova tuberculínica. Com perguntas subjetivas, o segundo eixo investigou saberes, opiniões e experiências sobre o tema para apreender as RS.

Esse instrumento não foi submetido a teste-piloto, mas foi avaliado por quatro professoras doutoras, vinculadas a Programas de Pós-Graduação em Enfermagem, selecionadas por terem notável produção intelectual e pesquisarem temas relacionados à educação superior, à enfermagem e à saúde coletiva. Entre elas, duas referendaram o instrumento e duas teceram sugestões, cujos aspectos pertinentes foram acatados para qualificá-lo. Tendo em vista a completude dos dados, não foi necessário repetir entrevistas ou incorporar outras técnicas de produção. Para não comprometer a espontaneidade dos depoimentos, as transcrições não foram compartilhadas com os participantes.

### Análise e Tratamento dos Dados

Os dados do perfil sociodemográfico e acadêmico foram tabulados em planilha do *Microsoft Office Excel*® (versão 2013) e analisados com estatística descritiva para evidenciar os números absolutos e relativos, destacando-se os resultados predominantes, exceto em relação às variáveis série/período semestral, situação conjugal e município de origem, pormenorizadas na íntegra. Os dados subjetivos foram transcritos para formar um *corpus* textual em arquivo único, sem formatação (arquivo do tipo .txt), importado ao *software* de acesso livre *Interface de R pour les Analyses Multidimensionnelles de Textes et de Questionnaires* (IRaMuTeQ®, versão 0.7, *alpha* 2), para realizar análise lexical. Por utilizar funções do *software* R, o IRaMuTeQ® gera dados estatísticos e semânticos a partir do processamento de fontes variadas, que tratam de tema único, como conjunto de entrevistas transcritas, auxiliando a identificar os seus significados^(13)^.

Cinco modalidades analíticas são ofertadas em seu menu: estatística simples (análise lexicográfica), especificidades e análise fatorial de correspondência, classificação hierárquica descendente (CHD), análise de similitude e nuvem de palavras. Entre elas, optou-se pela CHD, no intuito de formar classes lexicais que expressam o conteúdo do *corpus* e são ilustradas por dendrograma horizontal, que aponta a lógica de partição do *corpus* e os percentuais dos segmentos de texto (ST) que constituem as classes. As palavras associadas a elas são caracterizadas por quatro valores estatísticos: frequência de ST que contém a palavra na classe (Fc); frequência total de ST que contém a palavra no *corpus* (Ft); percentual (%) de ST que contém a palavra na classe, em relação à ocorrência no *corpus*; e qui-quadrado (X^2^), que demonstra a força associativa da palavra, considerando-a representativa quando p < 0,0001^(13)^.

Os dados foram interpretados à luz da literatura científica e dos processos cognitivos formadores das RS, que se baseiam na memória social e em conclusões passadas, e são conhecidos como ancoragem e objetivação. O primeiro consiste na familiarização de um objeto, inicialmente encarado como estranho e distinto da realidade, possibilitando classificá-lo e (re)nomeá- lo, de acordo com preceitos e valores do grupo. O segundo consiste na materialização do objeto por meio de imagem ou símbolo que visualmente o caracterize, motivos pelos quais as RS dependem das experiências individuais e coletivas, sendo toda representação constituída por dupla natureza, conceitual e figurativa, apontando para esses processos^(8)^.

### Aspectos Éticos

Foram atendidas as orientações da Resolução nº 466/2012 do Conselho Nacional de Saúde/Ministério da Saúde, obtendo- se autorização institucional e aprovação pelo Comitê de Ética em Pesquisa do Curso de Graduação em Enfermagem da universidade onde o estudo ocorreu, sob Parecer nº 5.458.024, emitido em junho de 2022. Todos os participantes leram e assinaram Termo de Consentimento Livre e Esclarecido, declarando o aceite, previamente às entrevistas. Para assegurar o sigilo de suas identidades, empregou-se código alfanumérico, constituído pela letra E, de “estudante”, seguida por algarismo romano (que indica a série do curso), hífen e número cardinal (que indica a ordem das entrevistas).

## RESULTADOS

### Perfil Sociodemográfico e Acadêmico dos Participantes

A idade variou entre 19 e 40 anos, destacando-se as faixas etárias de 19 a 23 (n = 28; 75,68%) e 24 a 28 anos (n = 6; 16,22%), sendo que 24 (64,86%) eram do sexo feminino. Quanto às séries e aos períodos semestrais, constavam: um (2,70%) cursando a 1ª série/bloco I e seis (16,22%) a 1ª série/bloco II, totalizando sete matriculados (18,92%) na 1ª série; dois (5,41%) cursavam a 2ª série/bloco I e três (8,11%) a 2ª série/bloco II, totalizando cinco matriculados (13,51%) na 2ª série; oito (21,62%) cursavam a 3ª série/bloco II; quatro (10,81%) cursavam a 4ª série/bloco I e seis (16,22%) a 4ª série/bloco II, totalizando 10 matriculados (27,03%) na 4ª série; quatro (10,81%) cursavam a 5ª série/bloco I e três (8,11%) a 5ª série/bloco II, totalizando sete matriculados (18,92%) na 5ª série.

Assim, a 3ª série/bloco I foi o único período do qual não houve participantes, devido à impossibilidade de pactuar dias e horários propícios às entrevistas. Todavia, isso não constituiu limitação, pois os outros períodos foram contemplados, caracterizando a participação efetiva de todas as séries.

Referente à cor/raça, 21 (56,76%) declararam-se pardos e 11 (29,73%) brancos. No tocante à religião, 17 (45,95%) eram evangélicos e 14 (37,84%) católicos. Tratando-se da situação conjugal, 32 (86,49%) eram solteiros, quatro (10,81%) viviam em união consensual e um (2,70%) era casado, sendo que a maioria (n = 35; 94,59%) afirmou não ter filhos. Identificou-se que 22 (59,46%) não recebiam bolsa de estudos e, entre os que recebiam, 14 bolsas eram provenientes de instituições públicas de ensino, assistência ou gestão em saúde, principalmente para estágio extracurricular (n = 7; 18,92%).

Além disso, 31 (83,78%) relataram não exercer atividades profissionais, 23 (62,16%) apresentavam renda familiar mensal entre dois e três salários mínimos, e oito (21,62%) apresentavam acima de cinco, considerando que, no Brasil, em 2023, o valor oficial do salário era de R$ 1.320,00. Quanto ao município de origem, 28 (75,68%) residiam em Belém e nove (24,32%) em outros municípios da Região Metropolitana. O número de pessoas com quem residiam variou entre um e 18, prevalecendo três pessoas (n = 13; 35,14%), sobretudo mãe, irmãos e pai, respectivamente mencionados por 25 (67,57%), 18 (48,65%) e 13 (35,14%) participantes. Acerca da prova tuberculínica, constatou-se que o tamanho da enduração variou entre cinco e 20 mm, predominando 10 (n = 13; 35,14%) e 15 (n = 7; 18,92%), com média aproximada de 11,70 mm.

### Apresentação dos Resultados do Corpus Textual

O IRaMuTeQ® identificou 37 textos, correspondentes ao conjunto das entrevistas. Por meio da CHD, os textos foram desmembrados em 1.939 ST, resultando no aproveitamento de 1.686 (86,95%), sendo identificadas 67.619 ocorrências (formas ou palavras), das quais 5.022 eram palavras distintas e 2.367 eram hápax (palavras com frequência igual a um), correspondendo a 47,13% das palavras distintas e 3,50% das ocorrências. A média de ocorrências por ST foi 34,87.

Mediante a lógica de partição do *corpus*, sete classes lexicais foram geradas, formando dois *subcorpora*, cujas classes apresentaram diferentes números absolutos e relativos de ST. Assim, o primeiro *subcorpus* constituiu-se pela classe 2 (com 281 ST; 16,67% do *corpus*), e o segundo, respectivamente pela classe 5 (175 ST; 10,38%), classe 6 (278 ST; 16,49%), classe 7 (277 ST; 16,43%), classe 1 (262 ST; 15,54%), classe 3 (174 ST; 10,32%) e classe 4 (239 ST; 14,18%), como evidencia o dendrograma horizontal da CHD, cujos percentuais foram arredondados, pelo IRaMuTeQ®, com uma casa decimal após a vírgula (Figura 1).

**Figura 1:** Dendrograma horizontal da classificação hierárquica descendente – Belém, Pará, Brasil, 2023.

Para atender ao objetivo deste estudo, optou-se por detalhar a classe 7, que emergiu do segundo *subcorpus* e apresentou 65 palavras representativas (p < 0,0001), entre as quais 21 (32,31%) destacaram-se, sobretudo por seus qui-quadrado, como mostra a Tabela 1.

**Tabela 1:** Detalhamento das palavras mais representativas da classe 7 – Belém, Pará, Brasil, 2023.

Nº	Palavras	Fc	Ft	%	X^2^
1	Informação	121	175	69,14	395,18
2	Curso	94	123	76,42	347,80
3	Falta	51	58	87,93	223,66
4	Assunto	57	84	67,86	170,29
5	Estudar	40	50	80,00	151,66
6	Componente_curricular	30	34	88,24	130,31
7	Componentes_curriculares	23	24	95,83	111,80
8	DIP	36	52	69,23	108,95
9	Estudantes_de_enfermagem	24	27	88,89	104,93
10	Semestre	18	19	94,74	85,82
11	Início	19	22	86,36	79,40
12	Microbiologia	13	13	100,00	66,64
13	Obter	15	17	88,24	64,49
14	Conhecimento	36	72	50,00	61,73
15	Ministrar	12	12	100,00	61,48
16	Oitavo_semestre	12	13	92,31	54,94
17	Antes	43	101	42,57	53,49
18	Estudante	26	47	55,32	53,26
19	Abordar	10	10	100,00	51,17
20	Aprofundado	12	14	85,71	49,36
21	Aprender	17	26	65,38	46,09

Nota: DIP: doenças infecciosas e parasitárias; Fc: frequência de ST que contém a palavra na classe; Ft: frequência total de ST que contém a palavra no *corpus*; %: percentual de ST que contém a palavra na classe, em relação à ocorrência no *corpus*; X^2^: qui-quadrado.

De acordo com seu conteúdo, essa classe foi nomeada pelos autores como “Informando sobre a ILTB” e organizada em um eixo temático, denominado “Informar para melhor cuidar: desafios e possibilidades na educação superior”, visando apresentar os dados subjetivos, que delinearam dois conjuntos representacionais.

Aborda-se a maneira como os participantes percebiam as condições em que as informações sobre a ILTB eram compartilhadas durante o curso de enfermagem, constituindo o primeiro conjunto representacional. Nesse sentido, apontaram sugestões que, na concepção deles, poderiam contribuir para solucionar os desafios inerentes a esse contexto, ou mitigar seus efeitos deletérios no cotidiano pessoal, acadêmico e profissional dos estudantes, constituindo o segundo conjunto representacional, como detalhado abaixo e exemplificado com alguns excertos emblemáticos.

### Eixo Temático – Informar Para Melhor Cuidar: Desafios e Possibilidades na Educação Superior

Os ST da classe 7 revelaram que o curso de graduação, vivenciado por eles, apresentava carência de informações qualificadas e oportunas sobre a ILTB, que fossem não somente apresentadas de maneira isolada, mas reiteradas e melhor fundamentadas no cotidiano acadêmico. Esse entendimento foi ancorado por dois fatores coexistentes, que limitavam a compreensão global do tema, considerando que os estudantes representaram a informação como elemento essencial para lidar com a ILTB.

O primeiro fator correspondeu à premissa de que, muitas vezes, o tema era introduzido tardiamente, por ocasião do componente curricular Enfermagem em Doenças Infecciosas e Parasitárias (DIP), no oitavo semestre do curso. Por sua vez, o segundo relacionou-se ao fato de que, independentemente do período letivo em que o tema era ministrado, muitas vezes sua apresentação ocorria em momento pontual, com superficialidade de conteúdo pelos professores, aparentemente não atribuindo a devida importância.

As duas palavras mais representativas, “informação” (X^2^ = 395,18) e “curso” (X^2^ = 347,80), associadas a palavras como “falta” (X^2^ = 223,66), “assunto” (X^2^ = 170,29), “DIP” (X^2^ = 108,95), “semestre” (X^2^ = 85,82), “microbiologia” (X^2^ = 66,64) e “aprofundado” (X^2^ = 49,36), ratificam esses fatores, fortemente ancorados na ideia de que configuram uma realidade negativa, razão pela qual foram objetivados por termos que remetem à superficialidade de conteúdo, como “muito raso” e “estilo ensino médio”, expressados por EIII-7:

*Acho que falta aprofundamento, pois a gente vê, por exemplo, em Microbiologia e em Patologia, mas achei muito raso, muito estilo ensino médio. Tanto que, se me perguntarem algo mais aprofundado sobre o assunto, não saberei responder e, particularmente, acho isso vergonhoso. Essa é tanto uma falha minha, por não ter aprofundado, quanto do ensino no curso. O que a gente teve foi uma aula* [de Microbiologia] *sobre isso, junto com vários outros assuntos, em uma manhã inteira, e uma apostila de um livro muito antigo.* (EIII-7)

*Acho que falta informação, porque, por exemplo, eu só descobri que existia esse teste* [prova tuberculínica] *porque realizei, mas não fazia ideia que existia essa condição, a ILTB. Falta dar mais visibilidade, porque a gente estuda sobre a TB, tem aula sobre o microrganismo, mas não se costuma falar da ILTB, que a gente pode ter a forma latente.* (EII-19)

*Antes da pesquisa* [referindo-se à testagem com a prova tuberculínica], *eu já tinha entrado em contato com paciente de TB, mas não tinha ideia concreta sobre a ILTB. Eu sabia o que era TB, como se realiza o tratamento, como se preenche a ficha de notificação, e conhecia alguns exames, mas, os detalhes, só soube semestres depois de entrar em contato com esse paciente* [conheceu detalhes no componente curricular Enfermagem em DIP]. (EIV-20)

*Falta informação sobre isso entre os estudantes, porque, naquele momento* [em que recebeu o resultado do teste], *eu não compreendia. Acho que ainda tenho dúvidas que preciso estudar e conversar com profissionais, até para não reforçar o estigma que existe sobre a doença e a ILTB.* (EIV-29)

Para superar os desafios que se impuseram frente à carência de informações, ou mitigar seus efeitos, os participantes teceram sugestões que atinam a dois aspectos principais: 1) necessidade de repensar e reestruturar a matriz curricular para que o tema seja ministrado previamente ao oitavo semestre, no componente curricular Enfermagem em DIP e/ou em outro(s) componente(s), com estratégias de ensino-aprendizagem pertinentes, ancorando-se no entendimento de que o oitavo semestre é tardio para introduzir o tema e que estratégias pouco interessantes podem dificultar o compartilhamento de informações; 2) promover diferentes espaços de diálogo e formação no ambiente universitário, objetivados sob os formatos de palestra e curso complementar, para esclarecer dúvidas e potencializar a disseminação do tema, considerando sua relevância.

Essas sugestões são corroboradas pela ocorrência dos verbos “estudar” (X^2^ = 151,66), “ministrar” (X^2^ = 61,48), “abordar” (X^2^ = 51,17) e “aprender” (X^2^ = 46,09), junto às palavras que indicam temporalidade, “início” (X^2^ = 79,40) e “antes” (X^2^ = 53,49). A primeira sugestão foi expressada, por exemplo, nos depoimentos de EIV-29 e EIII-34, e a segunda, nos de EIII-7 e EIII-25:

*Penso que a ILTB é uma condição que a gente estuda muito no final do curso, e poderíamos começar a discutir antes, por exemplo, em Antropologia, em que estudamos nossa relação com o outro. A universidade poderia trazer exemplos dentro de componentes curriculares como Antropologia e Filosofia, no contexto de pessoas doentes, para que a gente reforce o olhar sobre o doente e a transmissão da doença de forma mais ética e respeitosa e, assim, estimule o aprendizado.* (EIV-29)

*Conversando com pessoas que participaram do teste, percebi que muitas não conheciam a ILTB, não sabiam que ela existe. Na grade curricular do curso, deveria ter um componente curricular para abordar esse assunto mais cedo, nos primeiros semestres, pois não sei se ainda vou estudar. Mas deveria ter, porque a gente acaba vendo muito para frente, quando já passamos por vários cenários que tinham pessoas com TB, e essa situação é complicada.* (EIII-34)

*Isso poderia ser resolvido com mais cursos sobre o assunto. Por exemplo, fui para uma palestra sobre TB em determinado hospital, e foi muito bom, aprendi muitas coisas que não aprendi na escola e na universidade. Aprendi que não há só a TB pulmonar, pois a médica, que ministrou a palestra, falou sobre o adoecimento de vários outros órgãos.* (EIII-7)

*Acho que isso pode ser resolvido com palestras e troca de informações, não necessariamente em um semestre específico, mas desde o começo do curso, como no componente de Microbiologia.* (EIII-25)

## DISCUSSÃO

Como primeiro conjunto representacional, os resultados apresentam uma realidade delicada e preocupante, motivo pelo qual precisa ser revista pelas instâncias acadêmico-administrativas competentes. Segundo os participantes, essa realidade configurou-se no fato de que o curso apresentava limitações quanto ao compartilhamento oportuno de informações sobre a ILTB ao longo dos períodos letivos, culminando em fragilidades na formação acadêmica e profissional.

Sabe-se que as práticas de indivíduos e grupos, tais como as ações de cuidado consigo e com o outro, simbolizam a concretização das RS, posto que essa forma de conhecimento particular guia comunicações e comportamentos entre pessoas^(8)^. No entanto, é preciso que informações sobre um dado objeto de importância subjetiva sejam veiculadas, pois são importante elemento da elaboração das RS (dimensão da informação), como defendido por outros autores que realizaram estudos em interface com a TRS^(9,14)^.

Assim, é possível inferir que, sem informações adequadas, compartilhadas em momento oportuno e reiteradas em outros momentos do curso, os estudantes podem não se sentir capacitados ou seguros para realizar, com efetividade, seu autocuidado e as práticas de cuidado direcionadas aos grupos humanos, como os usuários dos serviços de saúde nos quais desenvolvem atividades supervisionadas.

Dialogando com essas evidências, destaca-se que, no contexto da formação acadêmica, estudo qualitativo, realizado com 25 estudantes do terceiro semestre do Curso de Graduação em Enfermagem de uma universidade pública no Sul do Brasil, analisou as contribuições do treinamento de habilidades por simulação, em uma disciplina do curso, como estratégia pedagógica para desenvolver competências. Entre outros resultados, o treinamento possibilitou fortalecer a destreza e a segurança para realizar procedimentos técnicos, previamente às aulas em cenários clínicos reais, fundamentando-se no conteúdo teórico e no *feedback* do professor quanto à execução dos procedimentos^(15)^.

Essa experiência oportunizou melhor administrar sentimentos, como ansiedade, insegurança e nervosismo, manifestos perante os desafios do treinamento. Além disso, reiterou conhecimentos, esclareceu dúvidas e estabeleceu atitudes de autoavaliação e autonomia intelectual, mobilizando os estudantes a se organizarem e tornarem corresponsáveis por seu aprendizado. Assim, com os ensinamentos do professor, reconheceram a necessidade de estudar para se aprofundar nos conhecimentos teórico-práticos da disciplina, influenciando diretamente a qualidade das ações de cuidado^(15)^.

Justificando as limitações de informação sobre a ILTB, os participantes ancoraram-nas em dois fatores. O primeiro fator correspondeu à introdução tardia do tema, no oitavo semestre do curso, e o segundo evidenciou que, apesar de sua importância, o tema era abordado de maneira isolada e superficial, fazendo com que os estudantes não o compreendessem adequadamente e não o relacionassem com outros conteúdos e/ou componentes curriculares, fragilizando o processo ensino-aprendizagem.

Na educação superior, a formação de recursos humanos para o campo da saúde exige que, para ser efetivo, esse processo seja crítico-reflexivo e dotado de experiências sistemáticas, principalmente na realidade do SUS. Tais experiências devem possibilitar a internalização de conteúdos e a criação de redes mentais que os associem de maneira coerente, no intuito de gerar sentidos que guiem as práticas profissionais^(16)^, contexto que sinaliza e reforça a natureza pragmática das RS.

Tratando-se da saúde humana, é preciso considerar que o indivíduo, a família e a comunidade são os sujeitos-alvo dessas práticas, fato justificado por serem complexos, multideterminados e não compartimentalizados. Assim, expressam características biopsicossociais, que devem ser valorizadas para que suas necessidades individuais e coletivas sejam atendidas de forma adequada^(17)^.

Esse contexto aponta para os cinco processos de trabalho em enfermagem (assistir, administrar, ensinar, pesquisar e participar politicamente) como aspectos que materializam a responsabilidade científica, ética, política e social da profissão^(18)^. Tais processos devem ser assumidos pelos enfermeiros no cotidiano de sua atuação, dependendo da natureza das atividades que desempenham com os grupos humanos. Por esse motivo, a educação superior deve capacitá-los para lidar com os desafios e as possibilidades da profissão.

No intuito de enfrentar as limitações quanto ao compartilhamento do tema, os estudantes desvelaram o segundo conjunto representacional, inerente às sugestões que, na concepção deles, se fossem implementadas, poderiam solucionar as limitações ou mitigar seus efeitos. Essas sugestões foram desdobradas em dois aspectos, que representam, em primeira instância, a necessidade de repensar/reestruturar a matriz curricular do curso, ancorada na ideia de compartilhar o tema em momento oportuno, e em segunda instância, estimular espaços de formação para dialogar sobre o tema, objetivados em diferentes formatos.

Assim, mostrou-se patente a ideia de somar esforços (espaços de formação), junto às atividades curriculares obrigatórias (previstas na matriz curricular), para esclarecer dúvidas e fortalecer o compartilhamento do tema com os estudantes, em diferentes períodos letivos, tendo em vista que poderiam ser expostos ao bacilo, por ocasião das atividades práticas, desde o início do curso. Essa representação encontra amparo na literatura, devido ao frequente contato desses atores com a comunidade e com cenários institucionais na saúde coletiva e na atenção hospitalar^(5–7)^.

Portanto, à luz dos resultados, infere-se que a possibilidade de exposição foi representada pelos estudantes como fator que justifica a necessidade de serem apresentados ao tema previamente ao oitavo semestre e discuti-lo ao longo do curso, no intuito de terem acesso a informações qualificadas que os auxiliem a desenvolver seu autocuidado, por meio das práticas de biossegurança para prevenir ILTB na comunidade e nos serviços de saúde, e desenvolver melhores práticas de cuidado direcionadas aos grupos humanos.

Em vista disso, é oportuno considerar que a realidade vivenciada pelos estudantes antagoniza o conceito ampliado de saúde, construído coletivamente na VIII Conferência Nacional de Saúde, realizada em 1986, e legitimado na Constituição Federal de 1988, contexto no qual a saúde passou a ser direito de todos os cidadãos e dever do Estado. Baseando-se nesse conceito, a Lei nº 8.080/1990, que regulamentou o SUS, declara que a saúde é multideterminada, pois resulta de condições ambientais, educacionais, habitacionais, nutricionais, ocupacionais, de lazer, liberdade, acesso à posse da terra e acesso aos serviços de saúde, entre outras^(19)^.

Essa concepção ampliada advém de um processo histórico, que envolve relações humanas e a capacidade de o homem refletir sobre a sua realidade^(20)^, constituindo não um mero capricho acadêmico, mas uma necessidade política^(21)^. Isso permite afirmar que, sem informações e processos educacionais de qualidade, o bem-estar do indivíduo e de sua coletividade pode ser fragilizado, posto que informação, conhecimento e comunicação se associam à promoção da saúde e à prevenção de doenças^(22)^, estabelecendo relações com o conceito ampliado de saúde^(17,19)^.

Os estudantes deixaram implícita a necessidade de os professores desenvolverem estratégias de ensino diferenciadas em seus componentes curriculares, no intuito de estimular o aprendizado sobre a ILTB. Entende-se que isso aponta para o uso adequado de metodologias ativas de aprendizagem, sendo necessário que a comunidade acadêmica reflita continuamente sobre a importância dessas metodologias, aplique-as e estimule-as, considerando o potencial que apresentam para formar recursos humanos qualificados. Assim, no campo da saúde, inclusive na educação em enfermagem, essas metodologias destacam-se na literatura nacional^(23,24)^ e internacional^(25,26)^.

Nessa perspectiva, ao tecerem a sugestão de as instâncias competentes promoverem espaços de formação sobre a ILTB, objetivados por cursos e palestras no ambiente universitário, os estudantes deixaram patente a vontade de se envolverem ativamente nesses espaços, participando e contribuindo para sua realização, como evidenciado pela expressão “troca de informações”, que aponta um processo dialógico.

Com base nas RS aqui desveladas e nas conexões que mantêm entre si, entende-se que as limitações de informação sobre a ILTB, na graduação em enfermagem, constituem força-motriz que obstaculiza, ao menos parcialmente, a consolidação do processo ensino-aprendizagem, com possíveis repercussões negativas na formação do enfermeiro, dependendo do grau das limitações.

Reiterando a importância da informação para as ações no contexto da TB, pesquisa metodológica com abordagem qualitativa, realizada com 41 enfermeiros que atuavam em unidades da APS no município de Belém, revelou a carência de conhecimentos sobre normas e diretrizes do Programa de Controle da TB entre as dificuldades, relatadas por eles, para controlar os casos da doença. Essas normas e diretrizes estavam relacionadas ao manejo dos casos, à indicação de exames laboratoriais e à necessidade de encaminhar o doente para a atenção secundária ou terciária, fragilizando as ações de cuidado e de vigilância epidemiológica^(27)^.

Para enfrentar esse cenário, sugeriram que uma tecnologia educacional, em formato documental, fosse elaborada e disponibilizada no cotidiano dos enfermeiros que atuam no controle da TB. Nesse sentido, apontaram a necessidade de conter informações que auxiliem o profissional a desenvolver suas atividades na APS, incluindo informações específicas sobre a ILTB, por entendê-la como agravo com características diferentes da TB. Essa pesquisa resultou na construção participativa de um guia instrutivo, visando atender peculiaridades locorregionais do cenário em que foi realizada^(27)^.

Identificando aproximações com outro tema, é perceptível que a carência de informações não ocorre somente com a ILTB. Como exemplo, cita-se uma revisão integrativa, com 10 estudos primários nacionais e internacionais, que analisou evidências sobre a assistência de enfermagem às crianças com necessidades especiais de saúde na APS. Entre outros resultados, demonstrou-se que os enfermeiros, em sua maioria, não se sentem capacitados para cuidar dessa população, em virtude de limitações na formação acadêmica e/ou nas capacitações profissionais regulares e específicas^(28)^.

Também é oportuno considerar que as limitações de informação antagonizam as Diretrizes Curriculares Nacionais (DCN) vigentes para o Curso de Graduação em Enfermagem, instituídas pela Resolução nº 3, de 7 de novembro de 2001, do Conselho Nacional de Educação^(29)^. Tal reflexão baseia-se no fato de que essas limitações constituem uma realidade contrária ao que as DCN orientam, isto é, a formação de profissionais com competências e habilidades técnico-científicas, éticas e humanísticas, conscientes de sua responsabilidade social, para atender às necessidades de saúde individuais e coletivas, visando mobilizar transformações na realidade dos grupos humanos.

No entanto, é preciso também situar a corresponsabilidade do estudante por sua formação, haja vista que deve empreender esforços possíveis para solucionar suas fragilidades de conhecimento e para fortalecer suas competências e habilidades. Com esse propósito, a educação superior deve estimular atitudes critico-reflexivas, por meio de processos emancipadores, que geram autonomia intelectual e, assim, mobilizam a busca ativa pelo conhecimento^(23)^.

Além disso, o processo ensino-aprendizagem deve ser construído coletivamente por professores e estudantes, razão pela qual a participação ativa de ambos é fundamental e intransferível^(25)^. Nesse sentido, as relações entre estudantes são igualmente importantes para estabelecer redes colaborativas, que os auxiliem a superar os desafios do processo ensino-aprendizagem^(30)^, a exemplo das fragilidades na compreensão de certos temas, como a ILTB, e de sua aplicação coerente nas práticas de autocuidado e nas práticas de cuidado direcionadas aos grupos humanos.

Como limitação deste estudo, menciona-se o fato de ter sido realizado no Curso de Graduação em Enfermagem de uma universidade pública em Belém, configurando cenário específico, o qual é influenciado por vários condicionantes, como fatores culturais, econômicos, políticos, sociais e territoriais. Isso caracteriza pequena representatividade geográfica dos dados, tendo em vista que esse município dispõe de outros Cursos de Graduação em Enfermagem, sobretudo vinculados a IES privadas, que não foram considerados devido às restrições impostas pela pandemia de COVID-19. Todavia, entende-se que as RS aqui desveladas podem, ao menos parcialmente, convergir com a realidade vivenciada/enfrentada por estudantes de enfermagem dessas e de outras IES nos cenários regional e nacional.

Assim, os resultados podem contribuir para que estudantes, professores e outros atores da comunidade acadêmica revisem/reestruturem matrizes curriculares e guiem processos de ensino-aprendizagem na educação superior, visando aperfeiçoar o compartilhamento de informações sobre a ILTB e qualificar a formação acadêmica e profissional. Além disso, podem contribuir para que instâncias universitárias competentes e autoridades públicas reflitam sobre a necessidade de fortalecer, na perspectiva da intersetorialidade entre os campos da saúde e da educação, as condições de biossegurança em serviços de saúde, no intuito de prevenir casos de ILTB nesse público, culminando por também fortalecer as ações de controle e de vigilância epidemiológica em torno desse agravo.

## CONCLUSÃO

Este estudo demonstrou que as RS de estudantes de enfermagem acerca do compartilhamento de informações sobre a ILTB delinearam dois conjuntos representacionais inerentes às suas experiências com o tema na educação superior. Tais conjuntos apontaram limitações que fragilizavam o compartilhamento oportuno e a qualidade das informações, além de sugestões que, na concepção dos estudantes, caso fossem implementadas, poderiam solucionar as limitações ou mitigar seus efeitos.

Perante esses resultados, salienta-se que, além das suas contribuições para a educação em enfermagem, a gestão acadêmica na educação superior e a gestão dos serviços de saúde nos quais os estudantes desenvolvem atividades práticas, este estudo pode contribuir para melhor compreender objetos ou fenômenos intersetoriais que envolvam saúde e educação, como o objeto psicossocial aqui investigado.

Nesse sentido, os resultados têm potencial para subsidiar a concepção teórico-metodológica de pesquisas que objetivem explorar outros aspectos relacionados à ILTB entre estudantes de enfermagem, valorizando a abordagem qualitativa, sobretudo em sua interface com a TRS, considerando o potencial dessa teoria para revelar, sistematizar e interpretar subjetividades no cotidiano de indivíduos e grupos, razão pela qual tem sido fortemente utilizada por pesquisadores da saúde e da educação.

## Data Availability

Os dados que dão suporte a este estudo podem ser disponibilizados mediante solicitação ao autor correspondente.

# Translation: Información sobre la infección tuberculosa latente en la educación superior: representaciones sociales de estudiantes de enfermería*

**DOI:** 10.1590/1980-220X-REEUSP-2025-0029es

**Authors:** Erlon Gabriel Rego de Andrade, Ivaneide Leal Ataíde Rodrigues, Laura Maria Vidal Nogueira, Eliza Paixão da Silva, Adriely Alciany Miranda dos Santos, Letícia Gabriela Noronha Rodrigues, Débora de Cássia Quaresma Silva, Vitória de Cássia Quaresma Silva

**Affiliations:** 1 Universidade do Estado do Pará, Escola de Enfermagem Magalhães Barata, Programa de Pós-Graduação em Enfermagem, Belém, PA, Brasil.; 2 Universidade do Estado do Pará, Centro de Ciências Biológicas e da Saúde, Escola de Enfermagem Magalhães Barata, Belém, PA, Brasil.; 3 Universidade Federal do Pará, Instituto de Ciências da Saúde, Faculdade de Enfermagem, Belém, PA, Brasil.

**Keywords:** Tuberculosis Latente, Estudiantes de Enfermería, Universidades, Representación Social, Psicología Social

## Abstract

**Objetivo:**

Analizar las representaciones sociales de estudiantes de enfermería sobre el intercambio de información sobre la infección tuberculosa latente en la educación superior.

**Método:**

Estudio descriptivo, cualitativo, anclado en el aspecto procedimental de la Teoría de las Representaciones Sociales. Se realizó con 37 estudiantes que reaccionaron a la prueba de la tuberculina, matriculados del 1º al 5º año, del Curso de Pregrado en Enfermería de una universidad pública de Belém, Pará, Brasil. Entre marzo y julio de 2023 se realizaron entrevistas individuales semiestructuradas, cuyo *corpus* fue sometido a análisis léxico con el programa *Interface de R pour les Analyses Multidimensionnelles de Textes et de Questionnaires* (0.7, *alpha* 2), utilizando clasificación jerárquica descendente.

**Resultados:**

Se identificaron 1.939 segmentos de texto, de los cuales 1.686 (86,95%) fueron utilizados, generándose siete clases léxicas. Se decidió detallar la clase 7, en la que se esquematizaron dos conjuntos de representación, abordando cómo se compartió la información y sugerencias sobre el tema.

**Conclusión:**

Las limitaciones que debilitaron el intercambio oportuno y la calidad de la información motivaron a los estudiantes a realizar sugerencias para solucionar los desafíos inherentes a este contexto o mitigar sus efectos perjudiciales.

## INTRODUCCIÓN

En la actualidad, la tuberculosis (TB) sigue representando un importante problema de salud pública, debido a la aparición de formas pulmonares y extrapulmonares, caracterizadas respectivamente por la afectación de los pulmones y otras estructuras anatomo-fisiológicas, marcadas por la multiplicación y propagación del *Mycobacterium tuberculosis*. Otra preocupación en el estado que precede a la enfermedad, conocido como infección tuberculosa latente (ITBL), que resulta de la exposición al bacilo expulsado por personas con TB pulmonar o TB laríngea sin tratamiento y se caracteriza por su instalación en los pulmones, generando una respuesta inmunológica adaptativa, sin desencadenar signos y síntomas, sin posibilidad de transmisión^(1,2)^.

Las personas inmunocompetentes que viven con el bacilo pueden no manifestar nunca la TB, pero las pruebas demuestran que el mayor riesgo de enfermedad se produce en los dos primeros años tras la infección, especialmente entre los grupos con vulnerabilidad inmunológica, como los menores de dos años o los mayores de 60, los que reciben terapia inmunosupresora, los que viven con el virus de la inmunodeficiencia humana (VIH) diabetes *mellitus* y/u otras enfermedades crónicas no transmisibles^(2)^. Las estimaciones indican que la ITBL afecta a aproximadamente una cuarta parte de la población mundial, lo que supone un gran contingente de personas susceptibles^(3)^.

La prueba tuberculínica es la prueba predominante para diagnosticar la ITBL en Brasil y es proporcionada por el Sistema Único de Salud (SUS). Requiere un profesional cualificado para aplicar, por vía intradérmica, 0,1 ml de tuberculina en el tercio medio de la cara anterior del antebrazo, lo que genera una reacción de hipersensibilidad cutánea, cuya endurecimiento debe medirse, considerando el diámetro transversal mayor. Se utiliza una regla milimetrada para leer e interpretar la prueba entre 48 y 72, o hasta 96 horas, después de la aplicación, debiéndose registrar el resultado en milímetros (mm), incluso cuando no haya endurecimiento (cero mm), considerándose reactivo (infectado) el individuo con endurecimiento igual o superior a cinco milímetros^(4)^.

Para desarrollar estrategias de control eficaces, en consonancia con la vigilancia epidemiológica, es necesario investigar la ITBL, sobre todo en los grupos que se caracterizan por una mayor exposición al bacilo, como los estudiantes de las áreas de la salud, entre los que destacan los estudiantes de enfermería, ya que los estudios han revelado una alta prevalencia de infección en este colectivo^(5,6)^. Dicha exposición se produce porque, durante el curso, estos estudiantes participan en actividades prácticas supervisadas, como las que se llevan a cabo en la Atención Primaria de Salud (APS), a través de programas de salud colectiva, con diferentes grupos humanos, entre los que destacan las personas con TB y sus acompañantes, cuidadores y familiares^(7)^.

Además de que los aspectos clínico-epidemiológicos son relevantes para comprender el tema, se entiende que los aspectos psicosociales atraviesan las experiencias de los estudiantes frente a las situaciones de exposición y al posible contagio, ya que, una vez infectados y conscientes de su condición a partir del diagnóstico, los estudiantes pueden alterar la forma en que comprenden su realidad y las relaciones que se tejen en ella con otros actores, como los usuarios de los servicios de salud, interiorizando la necesidad de emprender o transformar prácticas de autocuidado para evitar enfermarse, y prácticas de cuidado dirigidas a grupos humanos para atender sus necesidades biopsicosociales. Así, en la educación superior, el acceso oportuno a información de calidad sobre la ITBL durante la formación universitaria puede favorecer tales prácticas.

Esto apunta a la Teoría de las Representaciones Sociales (TRS), originaria de la psicología social y formulada por Serge Moscovici en la década de 1960. Su objetivo es comprender las representaciones sociales (RS), una forma de conocimiento que guía las estrategias con las que los individuos se comunican, interactúan y se comportan en sociedad^(8)^. Las RS establecen un lenguaje común que particulariza a los grupos frente a sus pertenencias sociales, es decir, características que señalan diferencias en las condiciones de producción de las RS^(9)^.

Presentan tres dimensiones básicas: la información, definida por los conocimientos sobre el objeto psicosocial y adquirida a través de las experiencias; la actitud, marcada por el juicio que el individuo hace sobre el objeto, induciéndole a una posición simbólica; y el campo de representación o dimensión de la imagen, que idealiza un modelo social, con elementos representativos dispuestos de manera ordenada y jerárquica. Por lo tanto, la información tiene un papel crucial en la producción de las RS y ayuda a comprender las prácticas cotidianas^(8)^.

Ante la relevancia del tema, se elaboró la pregunta orientadora: ¿cómo representan los estudiantes de enfermería el intercambio de información sobre la ITBL en la educación superior? Para responderla, este estudio se propuso analizar las representaciones sociales de los estudiantes de enfermería sobre el intercambio de información sobre la infección tuberculosa latente en la educación superior.

## MÉTODO

### Tipo de Estudio

Estudio descriptivo, con enfoque cualitativo, basado en la vertiente procesual de la TRS y guiado por el instrumento *Consolidated Criteria for Reporting Qualitative Research* (COREQ), considerando sus 32 ítems, distribuidos en tres dominios, inherentes al equipo de investigación, al diseño del estudio y al análisis y presentación de los resultados^(10)^. En consonancia con este enfoque, la vertiente procesal de la TRS permite comprender el proceso de construcción de las RS, revelando sus elementos constitutivos, que pueden identificarse, por ejemplo, a través de la expresión de aspectos cognitivos, ideológicos, imagéticos e informativos, o que denotan actitudes, creencias, opiniones y valores sociales^(9)^.

### Lugar

Se llevó a cabo en el curso de grado en Enfermería de una universidad pública en la ciudad de Belém, estado de Pará, Brasil. Con una duración de cinco años, el curso se imparte en cinco series y diez períodos semestrales (cada serie consta de dos bloques, identificados como bloque I y bloque II), con un total de 5000 horas de actividades teóricas y prácticas^(11)^. Este escenario fue elegido por sus características, que responden al interés de investigar el tema en el día a día de los estudiantes de enfermería.

### Población y Criterios de Selección

Participaron 37 estudiantes reactivos a la prueba tuberculínica, con un resultado igual o superior a cinco milímetros, matriculados regularmente y cursando entre el 1.º y el 5.º curso. Los estudiantes fueron previamente evaluados como parte de las actividades de una investigación multicéntrica, desarrollada en el marco del Programa Nacional de Cooperación Académica (PROCAD), ofrecido por la Coordinación de Perfeccionamiento del Personal de Nivel Superior (*Coordenação de Aperfeiçoamento de Pessoal de Nível Superior* – CAPES/Ministerio de Educación) para estimular redes colaborativas entre Programas de Posgrado *Stricto Sensu*, incluidos los programas de posgrado en enfermería. Esta investigación involucró a cuatro instituciones de educación superior (IES) públicas, ubicadas en las capitales de cuatro estados brasileños: Belém (Pará), Manaus (Amazonas), Río de Janeiro (Río de Janeiro) y Teresina (Piauí).

En Belém, las pruebas se realizaron entre diciembre de 2022 y febrero de 2023, periodo en el que había aproximadamente 345 estudiantes matriculados en el curso. Se realizaron pruebas a 269 (77,97% de adherencia), identificándose 74 reactores (27,51% de los testados), de los cuales se incluyeron 37 (50,00%) en este estudio. Este número cumplió con la recomendación de una muestra de 20 a 30 participantes, señalada en la literatura como empíricamente satisfactoria para alcanzar los objetivos en investigaciones cualitativas, y consideró la saturación de los datos, evidenciada cuando estos se mostraron suficientes y, por lo tanto, las adiciones no modificarían la comprensión de los autores sobre el objeto psicosocial^(12)^.

Dado que todos los que presentaron reacción eran elegibles, se optó por excluir a los que no pudieron ser localizados y a los que, aunque interesados, manifestaron imposibilidad de concertar una entrevista tras tres intentos, quedando excluidos 27 (36,49%), sin que se produjeran rechazos ni abandonos. Los demás (n = 10; 13,51%) no fueron contactados, ya que se cumplieron la recomendación muestral y la saturación de los datos.

### Recolección de Datos

Inicialmente, se presentó el proyecto de investigación a la coordinadora del curso para solicitar la autorización institucional y la cesión de una sala para realizar las entrevistas. Mediante autorización previa, concedida por los estudiantes, el equipo de investigadores involucrados en la prueba puso a disposición del primer autor una lista con los nombres, contactos e información específica de la prueba de los reactores. Así, el primer autor se puso en contacto con ellos a través de una aplicación de mensajería instantánea o por teléfono, momento en el que les proporcionó información sobre la investigación para invitarles a participar.

Con los que aceptaron, se programó una reunión en la sala reservada con el fin de detallar y aclarar los objetivos, procedimientos, riesgos y beneficios, obtener el consentimiento formal/voluntario y realizar entrevistas individuales, técnica de producción que se ajusta al aspecto procesal de la TRS^(9)^. Dado que el primer autor formaba parte del equipo de pruebas y se encargaba de las actividades para permitir la aplicación, lectura e interpretación de la prueba tuberculínica, los estudiantes lo conocían previamente y estaban informados sobre su trayectoria y sus aspiraciones académicas/profesionales, incluido el hecho de que este estudio era un requisito obligatorio para completar el máster en enfermería.

Las entrevistas fueron realizadas por el primer autor entre marzo y julio de 2023, previamente capacitado en reuniones de orientación, que formaban parte de las actividades curriculares del posgrado *stricto sensu*, y en reuniones periódicas de un grupo de investigación vinculado a la institución de los autores. Para garantizar la comodidad y la privacidad, y siguiendo algunas recomendaciones sanitarias para controlar la pandemia de COVID-19, aún vigentes en ese momento, solo el participante y el primer autor ocupaban la sala, que se mantenía ventilada y disponía de gel hidroalcohólico al 70%, informándose de la posibilidad de utilizar mascarilla, aunque este procedimiento ya no era obligatorio en el momento en que se recopilaron los datos. Las entrevistas tuvieron una duración mínima de 20 y máxima de 50 minutos, y se grabaron en formato MP3 con un dispositivo electrónico.

Para orientarlas, se elaboró un guion semiestructurado, compuesto por dos ejes, uno con preguntas para conocer el perfil sociodemográfico y académico de los participantes, demostrando su pertenencia social^(9)^, el que investigó las variables edad, sexo, curso y semestre del curso, color/raza, religión, situación conyugal, número de hijos, recepción de becas y institución de fomento, ocupación, renta familiar mensual, municipio de origen, número de personas con las que residían y quiénes eran esas personas, además del tamaño de la endurecimiento generado por la prueba tuberculínica. Utilizando preguntas subjetivas, el segundo eje investigó conocimientos, opiniones y experiencias sobre el tema para comprender las RS.

Este instrumento no se sometió a una prueba piloto, pero fue evaluado por cuatro profesoras doctoras, vinculadas a programas de posgrado en enfermería, seleccionadas por su notable producción intelectual y por investigar temas relacionados con la educación superior, la enfermería y la salud colectiva. Entre ellas, dos refrendaron el instrumento y dos hicieron sugerencias, cuyos aspectos pertinentes fueron aceptados para calificarlo. Teniendo en cuenta la exhaustividad de los datos, no fue necesario repetir las entrevistas ni incorporar otras técnicas de producción. Para no comprometer la espontaneidad de los testimonios, las transcripciones no se compartieron con los participantes.

### Análisis y Tratamiento de Los Datos

Los datos sociodemográficos y académicos se tabularon en una hoja de cálculo de *Microsoft Office Excel*® (versión 2013) y se analizaron con estadísticas descriptivas para evidenciar los números absolutos y relativos, destacando los resultados predominantes, excepto en relación con las variables serie/período semestral, situación conyugal y municipio de origen, que se detallan en su totalidad. Los datos subjetivos se transcribieron para formar un *corpus* textual en un único archivo, sin formato (archivo de tipo .txt), importado al *software* de acceso libre *Interface de R pour les Analyses Multidimensionnelles de Textes et de Questionnaires* (IRaMuTeQ®, versión 0.7, *alpha* 2), para realizar el análisis léxico. Al utilizar funciones del *software* R, IRaMuTeQ® genera datos estadísticos y semánticos a partir del procesamiento de fuentes variadas que tratan un tema único, como un conjunto de entrevistas transcritas, lo que ayuda a identificar sus significados^(13)^.

En su menú se ofrecen cinco modalidades analíticas: estadística simple (análisis lexicográfico), especificidades y análisis factorial de correspondencias, clasificación jerárquica descendente (CJD), análisis de similitud y nube de palabras. Entre ellas, se optó por la CJD, con el fin de formar clases léxicas que expresan el contenido del *corpus* y se ilustran mediante un dendrograma horizontal, que señala la lógica de partición del *corpus* y los porcentajes de los segmentos de texto (ST) que constituyen las clases. Las palabras asociadas a ellas se caracterizan por cuatro valores estadísticos: frecuencia de ST que contiene la palabra en la clase (Fc); frecuencia total de ST que contiene la palabra en el *corpus* (Ft); porcentaje (%) de ST que contiene la palabra en la clase, en relación con la ocurrencia en el *corpus*; y chi-cuadrado (X^2^), que demuestra la fuerza asociativa de la palabra, considerándola representativa cuando p < 0,0001^(13)^.

Los datos se interpretaron a la luz de la literatura científica y de los procesos cognitivos que conforman las RS, basados en la memoria social y en conclusiones pasadas, y conocidos como anclaje y objetivación. El primero consiste en familiarizarse con un objeto, inicialmente considerado extraño y distinto de la realidad, lo que permite clasificarlo y (re)nombrarlo, de acuerdo con los preceptos y valores del grupo. El segundo consiste en la materialización del objeto mediante una imagen o símbolo que lo caracterice visualmente, por lo que las RS dependen de las experiencias individuales y colectivas, y toda representación tiene una doble naturaleza, conceptual y figurativa, que apunta a estos procesos^(8)^.

### Aspectos Éticos

Se cumplieron las directrices de la Resolución n.º 466/2012 del Consejo Nacional de Salud/Ministerio de Salud, obteniéndose la autorización institucional y la aprobación del Comité de Ética en Investigación del Curso de Graduación en Enfermería de la universidad donde se realizó el estudio, mediante el Dictamen n.º 5.458.024, emitido en junio de 2022. Todos los participantes leyeron y firmaron un Término de Consentimiento Libre y Esclarecido, en el que declaraban su aceptación, antes de las entrevistas. Para garantizar el anonimato de sus identidades, se utilizó un código alfanumérico, compuesto por la letra E, de «estudiante», seguida de un número romano (que indica el curso), un guion y un número cardinal (que indica el orden de las entrevistas).

## RESULTADOS

### Perfil Sociodemográfico y Académico de Los Participantes

La edad osciló entre los 19 y los 40 años, destacando los grupos de edad de 19 a 23 (n = 28; 75,68%) y de 24 a 28 años (n = 6; 16,22%), siendo 24 (64,86%) del sexo femenino. En cuanto a los cursos y semestres, se observó lo siguiente: uno (2,70%) cursaba el 1.º curso/bloque I y seis (16,22%) el 1.º curso/bloque II, lo que sumaba un total de siete matriculados (18,92%) en el 1.º curso; dos (5,41%) cursaban el 2.º curso/bloque I y tres (8,11%) el 2.º curso/bloque II, lo que totalizaba cinco matriculados (13,51%) en 2.º curso; ocho (21,62%) cursaban 3.º curso/bloque II; cuatro (10,81%) cursaban 4.º curso/bloque I y seis (16,22%) 4.º curso/bloque II, lo que totalizaba 10 matriculados (27,03%) en 4.º curso; cuatro (10,81%) cursaban 5.º curso/bloque I y tres (8,11%) cursaban 5.º curso/bloque II, con un total de siete matriculados (18,92%) en 5.º curso.

Así, el 3.º curso/bloque I fue el único periodo en el que no hubo participantes, debido a la imposibilidad de acordar días y horarios adecuados para las entrevistas. Sin embargo, esto no supuso una limitación, ya que se contemplaron los demás periodos, lo que caracterizó la participación efectiva de todos los cursos.

En cuanto al color/raza, 21 (56,76%) se declararon pardos y 11 (29,73%) blancos. En cuanto a la religión, 17 (45,95%) eran evangélicos y 14 (37,84%) católicos. En cuanto al estado civil, 32 (86,49%) eran solteros, cuatro (10,81%) vivían en unión libre y uno (2,70%) estaba casado, y la mayoría (n = 35; 94,59%) afirmó no tener hijos. Se identificó que 22 (59,46%) no recibían becas de estudio y, entre los que sí las recibían, 14 becas procedían de instituciones públicas de enseñanza, asistencia o gestión en salud, principalmente para prácticas extracurriculares (n = 7; 18,92%).

Además, 31 (83,78%) declararon no ejercer ninguna actividad profesional, 23 (62,16%) tenían unos ingresos familiares mensuales de entre dos y tres salarios mínimos, y ocho (21,62%) tenían más de cinco, teniendo en cuenta que, en Brasil, en 2023, el salario oficial era de 1320,00 reales. En cuanto al municipio de origen, 28 (75,68%) residían en Belém y nueve (24,32%) en otros municipios de la Región Metropolitana. El número de personas con las que residían variaba entre una y 18, prevaleciendo tres personas (n = 13; 35,14%), sobre todo la madre, los hermanos y el padre, mencionados respectivamente por 25 (67,57%), 18 (48,65%) y 13 (35,14%) participantes. En cuanto a la prueba tuberculínica, se observó que el tamaño de la endurecimiento variaba entre cinco y 20 mm, predominando 10 (n = 13; 35,14%) y 15 (n = 7; 18,92%), con una media aproximada de 11,70 mm.

### Presentación de Los Resultados del Corpus Textual

El IRaMuTeQ® identificó 37 textos, correspondientes al conjunto de entrevistas. Mediante la CJD, los textos se dividieron en 1.939 ST, lo que resultó en el aprovechamiento de 1.686 (86,95%), identificándose 67.619 ocurrencias (formas o palabras), de las cuales 5.022 eran palabras distintas y 2.367 eran hapax (palabras con frecuencia igual a uno), lo que corresponde al 47,13% de las palabras distintas y al 3,50% de las ocurrencias. La media de ocurrencias por ST fue de 34,87.

Mediante la lógica de partición del *corpus*, se generaron siete clases léxicas, formando dos *subcorpora*, cuyas clases presentaron diferentes números absolutos y relativos de ST. Así, el primer *subcorpus* estaba constituido por la clase 2 (con 281 ST; 16,67% del *corpus*), y el segundo, respectivamente, por la clase 5 (175 ST; 10,38%), la clase 6 (278 ST; 16,49%), la clase 7 (277 ST; 16,43%), clase 1 (262 ST; 15,54%), clase 3 (174 ST; 10,32%) y clase 4 (239 ST; 14,18%), como lo evidencia el dendrograma horizontal de la CJD, cuyos porcentajes fueron redondeados por IRaMuTeQ® con un decimal después de la coma (Figura 1).

**Figura 1:** Dendrograma horizontal de la clasificación jerárquica descendente – Belém, Pará, Brasil, 2023.

Nota: *classe* = clase.

Para cumplir el objetivo de este estudio, optamos por detallar la clase 7, surgida del segundo *subcorpus* y que presentaba 65 palabras representativas (p < 0,0001), entre las que destacaban 21 (32,31%), sobre todo por su chi-cuadrado, como se muestra en la Tabla 1.

**Tabla 1:** Desglose de las palabras más representativas de la clase 7 – Belém, Pará, Brasil, 2023.

No.	Palabras	Fc	Ft	%	X^2^
1	Información	121	175	69,14	395,18
2	Curso	94	123	76,42	347,80
3	Falta	51	58	87,93	223,66
4	Asunto	57	84	67,86	170,29
5	Estudiar	40	50	80,00	151,66
6	Componente_curricular	30	34	88,24	130,31
7	Componentes_curriculares	23	24	95,83	111,80
8	EIP	36	52	69,23	108,95
9	Estudiantes_de_enfermería	24	27	88,89	104,93
10	Semestre	18	19	94,74	85,82
11	Inicio	19	22	86,36	79,40
12	Microbiología	13	13	100,00	66,64
13	Obtener	15	17	88,24	64,49
14	Conocimientos	36	72	50,00	61,73
15	Impartir	12	12	100,00	61,48
16	Octavo_semestre	12	13	92,31	54,94
17	Antes	43	101	42,57	53,49
18	Estudiante	26	47	55,32	53,26
19	Acercarse	10	10	100,00	51,17
20	En_profundidad	12	14	85,71	49,36
21	Aprender	17	26	65,38	46,09

Nota: EIP: enfermedades infecciosas y parasitarias; Fc: frecuencia de ST que contienen la palabra en la clase; Ft: frecuencia total de ST que contienen la palabra en el *corpus*; %: porcentaje de ST que contienen la palabra en la clase, en relación con la ocurrencia en el *corpus*; X^2^: chi-cuadrado.

De acuerdo con su contenido, los autores denominaron esta clase «Informar sobre la ITBL» y la organizaron en un eje temático denominado «Informar para cuidar mejor: retos y posibilidades en la educación superior», con el objetivo de presentar los datos subjetivos, que delinearon dos conjuntos representativos.

Se aborda la forma en que los participantes percibían las condiciones en las que se compartía la información sobre la ITBL durante el curso de enfermería, lo que constituye el primer conjunto representativo. En este sentido, señalaron sugerencias que, en su opinión, podrían contribuir a resolver los retos inherentes a este contexto o mitigar sus efectos perjudiciales en la vida personal, académica y profesional de los estudiantes, constituyendo el segundo conjunto representativo, como se detalla a continuación y se ejemplifica con algunos extractos emblemáticos.

### Eje Temático – Informar Para Cuidar Mejor: Retos y Posibilidades en la Educación Superior

Los ST de la clase 7 revelaron que el curso de grado que cursaban presentaba una falta de información cualificada y oportuna sobre la ITBL, que no solo se presentaba de forma aislada, sino que se reiteraba y estaba mejor fundamentada en el día a día académico. Esta percepción se anclaba en dos factores coexistentes que limitaban la comprensión global del tema, teniendo en cuenta que los estudiantes consideraban la información como un elemento esencial para abordar la ITBL.

El primer factor correspondía a la premisa de que, a menudo, el tema se introducía tardíamente, en el componente curricular Enfermería en Enfermedades Infecciosas y Parasitarias (EIP), en el octavo semestre del curso. Por su parte, el segundo se relacionaba con el hecho de que, independientemente del período lectivo en que se impartía el tema, a menudo su presentación se realizaba en un momento puntual, con un contenido superficial por parte de los profesores, que aparentemente no le atribuían la importancia debida.

Las dos palabras más representativas, «información» (X^2^ = 395,18) y «curso» (X^2^ = 347,80), asociadas a palabras como «falta» (X^2^ = 223,66), «asunto» (X^2^ = 170,29), «EIP» (X^2^ = 108,95), «semestre» (X^2^ = 85,82), «microbiología» (X^2^ = 66,64) y «en profundidad» (X^2^ = 49,36), ratifican estos factores, fuertemente anclados en la idea de que configuran una realidad negativa, razón por la cual fueron objetivados por términos que remiten a la superficialidad del contenido, como «muy superficial» y «estilo secundario», expresados por EIII-7:

*Creo que falta profundidad, porque vemos, por ejemplo, en Microbiología y en Patología, pero me pareció muy superficial, muy estilo secundario. Tanto es así que, si me preguntan algo más profundo sobre el tema, no sabré responder y, particularmente, me parece vergonzoso. Esto es tanto un fallo mío, por no haber profundizado, como de la enseñanza en el curso. Lo que tuvimos fue una clase* [de Microbiología] *sobre eso, junto con varios otros asuntos, durante toda una mañana, y un folleto de un libro muy antiguo.* (EIII-7)

*Creo que falta información, porque, por ejemplo, yo solo descubrí que existía esa prueba* [prueba tuberculínica] *porque me la hice, pero no tenía ni idea de que existía esa enfermedad, la ITBL. Hay que darle más visibilidad, porque estudiamos sobre la TB, tenemos clases sobre el microorganismo, pero no se suele hablar de la ITBL, que podemos tener de forma latente.* (EII-19)

*Antes de la investigación* [refiriéndose a la prueba tuberculínica], *ya había estado en contacto con pacientes con TB, pero no tenía una idea concreta sobre la ITBL. Sabía lo que era la TB, cómo se realiza el tratamiento, cómo se rellena el formulario de notificación y conocía algunos exámenes, pero los detalles solo los supe semestres después de entrar en contacto con este paciente* [conoció los detalles en el componente curricular Enfermería en EIP]. (EIV-20)

*Hay falta de información al respecto entre los estudiantes, porque en ese momento* [cuando recibió el resultado de la prueba], *yo no lo entendía. Creo que todavía tengo dudas que necesito estudiar y conversar con profesionales, incluso para no reforzar el estigma que existe sobre la enfermedad y la ITBL.* (EIV-29)

Para superar los retos que se plantearon ante la falta de información, o mitigar sus efectos, los participantes formularon sugerencias que se refieren a dos aspectos principales: 1) necesidad de repensar y reestructurar la matriz curricular para que el tema se imparta antes del octavo semestre, en el componente curricular Enfermería en EIP y/u otro(s) componente(s), con estrategias de enseñanza-aprendizaje pertinentes, anclándose en el entendimiento de que el octavo semestre es tarde para introducir el tema y que las estrategias poco interesantes pueden dificultar el intercambio de información; 2) promover diferentes espacios de diálogo y formación en el entorno universitario, objetivados en los formatos de conferencias y cursos complementarios, para aclarar dudas y potenciar la difusión del tema, teniendo en cuenta su relevancia.

Estas sugerencias se ven corroboradas por la aparición de los verbos «estudiar» (X^2^ = 151,66), «impartir» (X^2^ = 61,48), «acercarse» (X^2^ = 51,17) y «aprender» (X^2^ = 46,09), junto con palabras que indican temporalidad, «inicio» (X^2^ = 79,40) y «antes» (X^2^ = 53,49). La primera sugerencia se expresó, por ejemplo, en los testimonios de EIV-29 y EIII-34, y la segunda, en los de EIII-7 y EIII-25:

*Creo que la ITBL es una condición que estudiamos mucho al final del curso, y podríamos empezar a discutir antes, por ejemplo, en Antropología, donde estudiamos nuestra relación con el otro. La universidad podría aportar ejemplos dentro de componentes curriculares como Antropología y Filosofía, en el contexto de personas enfermas, para que reforcemos la mirada sobre el enfermo y la transmisión de la enfermedad de una forma más ética y respetuosa y, así, estimular el aprendizaje.* (EIV-29)

*Hablando con personas que participaron en la prueba, me di cuenta de que muchas no conocían la ITBL, no sabían que existía. En el plan de estudios del curso debería haber un componente curricular para abordar este tema más temprano, en los primeros semestres, porque no sé si voy a seguir estudiando. Pero debería haberlo, porque acabamos viendo mucho más adelante, cuando ya hemos pasado por varias situaciones en las que había personas con TB, y esa situación es complicada.* (EIII-34)

*Esto podría resolverse con más cursos sobre el tema. Por ejemplo, fui a una conferencia sobre la tuberculosis en un hospital y fue muy buena, aprendí muchas cosas que no había aprendido en la escuela ni en la universidad. Aprendí que no solo existe la tuberculosis pulmonar, ya que la médica que impartió la conferencia habló sobre la enfermedad en varios otros órganos.* (EIII-7)

*Creo que esto se puede resolver con charlas e intercambio de información, no necesariamente en un semestre específico, sino desde el comienzo del curso, como en la asignatura de Microbiología.* (EIII-25)

## DISCUSIÓN

Como primer conjunto representativo, los resultados presentan una realidad delicada y preocupante, por lo que debe ser revisada por las instancias académicas y administrativas competentes. Según los participantes, esta realidad se configuró en el hecho de que el curso presentaba limitaciones en cuanto al intercambio oportuno de información sobre la ITBL a lo largo de los períodos lectivos, lo que culminó en deficiencias en la formación académica y profesional.

Se sabe que las prácticas de individuos y grupos, tales como las acciones de cuidado de uno mismo y de los demás, simbolizan la concretización de las RS, ya que esta forma de conocimiento particular guía las comunicaciones y los comportamientos entre las personas^(8)^. Sin embargo, es necesario que se transmita la información sobre un objeto de importancia subjetiva, ya que es un elemento importante en la elaboración de las RS (dimensión de la información), como defienden otros autores que han realizado estudios relacionados con la TRS^(9,14)^.

Así, se puede inferir que, sin información adecuada, compartida en el momento oportuno y reiterada en otros momentos del curso, los estudiantes pueden no sentirse capacitados o seguros para realizar con eficacia su autocuidado y las prácticas de cuidado dirigidas a grupos humanos, como los usuarios de los servicios de salud en los que desarrollan actividades supervisadas.

En diálogo con estas evidencias, cabe destacar que, en el contexto de la formación académica, un estudio cualitativo realizado con 25 estudiantes del tercer semestre del curso de grado en Enfermería de una universidad pública del sur de Brasil analizó las contribuciones de la formación en habilidades mediante simulación, en una asignatura del curso, como estrategia pedagógica para desarrollar competencias. Entre otros resultados, la formación permitió reforzar la destreza y la seguridad para realizar procedimientos técnicos, antes de las clases en escenarios clínicos reales, basándose en el contenido teórico y en el *feedback* del profesor sobre la ejecución de los procedimientos^(15)^.

Esta experiencia permitió gestionar mejor sentimientos como la ansiedad, la inseguridad y el nerviosismo, manifestados ante los retos de la formación. Además, reafirmó conocimientos, aclaró dudas y estableció actitudes de autoevaluación y autonomía intelectual, movilizando a los estudiantes a organizarse y a hacerse corresponsables de su aprendizaje. Así, con las enseñanzas del profesor, reconocieron la necesidad de estudiar para profundizar en los conocimientos teórico-prácticos de la disciplina, lo que influyó directamente en la calidad de las acciones de cuidado^(15)^.

Los participantes justificaron las limitaciones de información sobre la ITBL anclándose en dos factores. El primer factor correspondió a la introducción tardía del tema, en el octavo semestre del curso, y el segundo puso de manifiesto que, a pesar de su importancia, el tema se abordaba de manera aislada y superficial, lo que hacía que los estudiantes no lo comprendieran adecuadamente y no lo relacionaran con otros contenidos y/o componentes curriculares, lo que debilitaba el proceso de enseñanza-aprendizaje.

En la educación superior, la formación de recursos humanos para el campo de la salud exige que, para ser eficaz, este proceso sea crítico-reflexivo y dotado de experiencias sistemáticas, principalmente en la realidad del SUS. Dichas experiencias deben permitir la internalización de contenidos y la creación de redes mentales que los asocien de manera coherente, con el fin de generar significados que guíen las prácticas profesionales^(16)^, contexto que señala y refuerza la naturaleza pragmática de las RS.

Al tratarse de la salud humana, es necesario considerar que el individuo, la familia y la comunidad son los sujetos destinatarios de estas prácticas, hecho justificado por su complejidad, multideterminación y no compartimentación. Así, expresan características biopsicosociales que deben valorarse para que sus necesidades individuales y colectivas sean atendidas de manera adecuada^(17)^.

Este contexto apunta a los cinco procesos de trabajo en enfermería (asistir, administrar, enseñar, investigar y participar políticamente) como aspectos que materializan la responsabilidad científica, ética, política y social de la profesión^(18)^. Dichos procesos deben ser asumidos por los enfermeros en el día a día de su actuación, dependiendo de la naturaleza de las actividades que realizan con los grupos humanos. Por esta razón, la educación superior debe capacitarles para hacer frente a los retos y posibilidades de la profesión.

Con el fin de hacer frente a las limitaciones en cuanto a la puesta en común del tema, los estudiantes revelaron el segundo conjunto representativo, inherente a las sugerencias que, en su opinión, si se aplicaran, podrían resolver las limitaciones o mitigar sus efectos. Estas sugerencias se desglosaron en dos aspectos, que representan, en primer lugar, la necesidad de repensar/reestructurar la matriz curricular del curso, anclada en la idea de compartir el tema en el momento oportuno y, en segundo lugar, estimular espacios de formación para dialogar sobre el tema, objetivados en diferentes formatos.

Así, se puso de manifiesto la idea de sumar esfuerzos (espacios de formación), junto con las actividades curriculares obligatorias (previstas en la matriz curricular), para aclarar dudas y fortalecer el intercambio del tema con los estudiantes, en diferentes períodos lectivos, teniendo en cuenta que podrían estar expuestos al bacilo, con ocasión de las actividades prácticas, desde el inicio del curso. Esta representación encuentra respaldo en la literatura, debido al contacto frecuente de estos actores con la comunidad y con escenarios institucionales en la salud colectiva y la atención hospitalaria^(5-7)^.

Por lo tanto, a la luz de los resultados, se deduce que la posibilidad de exposición fue representada por los estudiantes como un factor que justifica la necesidad de presentarles el tema antes del octavo semestre y discutirlo a lo largo del curso, con el fin de que tengan acceso a información calificada que les ayude a desarrollar su autocuidado, mediante prácticas de bioseguridad para prevenir la ITBL en la comunidad y en los servicios de salud, y desarrollar mejores prácticas de cuidado dirigidas a los grupos humanos.

En vista de ello, es oportuno considerar que la realidad vivida por los estudiantes se opone al concepto ampliado de salud, construido colectivamente en la VIII Conferencia Nacional de Salud, celebrada en 1986, y legitimado en la Constitución Federal de 1988, contexto en el que la salud pasó a ser un derecho de todos los ciudadanos y un deber del Estado. Basándose en este concepto, la Ley n.º 8.080/1990, que reguló el SUS, declara que la salud es multideterminada, ya que es el resultado de condiciones ambientales, educativas, de vivienda, nutricionales, ocupacionales, de ocio, libertad, acceso a la propiedad de la tierra y acceso a los servicios de salud, entre otras^(19)^.

Esta concepción ampliada proviene de un proceso histórico que involucra las relaciones humanas y la capacidad del hombre para reflexionar sobre su realidad^(20)^, constituyendo no un mero capricho académico, sino una necesidad política^(21)^. Esto permite afirmar que, sin información y procesos educativos de calidad, el bienestar del individuo y de su colectividad puede verse fragilizado, ya que la información, el conocimiento y la comunicación se asocian a la promoción de la salud y a la prevención de enfermedades^(22)^, estableciendo relaciones con el concepto ampliado de salud^(17,19)^.

Los estudiantes dejaron implícita la necesidad de que los profesores desarrollen estrategias de enseñanza diferenciadas en sus componentes curriculares, con el fin de estimular el aprendizaje sobre la ITBL. Se entiende que esto apunta al uso adecuado de metodologías activas de aprendizaje, siendo necesario que la comunidad académica reflexione continuamente sobre la importancia de estas metodologías, las aplique y las estimule, considerando el potencial que presentan para formar recursos humanos calificados. Así, en el campo de la salud, incluida la educación en enfermería, estas metodologías destacan en la literatura nacional^(23,24)^ e internacional^(25,26)^.

En esta perspectiva, al sugerir que las instancias competentes promuevan espacios de formación sobre la ITBL, objetivados en cursos y conferencias en el ámbito universitario, los estudiantes dejaron patente su voluntad de participar activamente en estos espacios, contribuyendo a su realización, como lo evidencia la expresión «intercambio de información», que apunta a un proceso dialógico.

Sobre la base de las RS aquí reveladas y de las conexiones que mantienen entre sí, se entiende que las limitaciones de información sobre la ITBL en la licenciatura en enfermería constituyen una fuerza motriz que obstaculiza, al menos parcialmente, la consolidación del proceso de enseñanza-aprendizaje, con posibles repercusiones negativas en la formación del enfermero, dependiendo del grado de las limitaciones.

Reiterando la importancia de la información para las acciones en el contexto de la TB, una investigación metodológica con enfoque cualitativo, realizada con 41 enfermeros que trabajaban en unidades de APS en el municipio de Belém, reveló la falta de conocimientos sobre las normas y directrices del Programa de Control de la TB entre las dificultades que ellos mismos relataron para controlar los casos de la enfermedad. Estas normas y directrices estaban relacionadas con el manejo de los casos, la indicación de exámenes de laboratorio y la necesidad de derivar al paciente a la atención secundaria o terciaria, lo que debilitaba las acciones de atención y vigilancia epidemiológica^(27)^.

Para hacer frente a esta situación, sugirieron que se elaborara una tecnología educativa, en formato documental, y se pusiera a disposición de los enfermeros que trabajan en el control de la TB en su día a día. En este sentido, señalaron la necesidad de incluir información que ayudara al profesional a desarrollar sus actividades en la APS, incluyendo información específica sobre la ITBL, al entenderla como una afección con características diferentes a la TB. Esta investigación dio lugar a la elaboración participativa de una guía instructiva, con el objetivo de atender las peculiaridades locorregionales del escenario en el que se llevó a cabo^(27)^.

Al identificar similitudes con otro tema, se observa que la falta de información no se limita a la ITBL. Como ejemplo, cabe citar una revisión integradora, con 10 estudios primarios nacionales e internacionales, que analizó las evidencias sobre la asistencia de enfermería a niños con necesidades especiales de salud en la APS. Entre otros resultados, se demostró que la mayoría de los enfermeros no se sienten capacitados para atender a esta población, debido a las limitaciones en su formación académica y/o en la capacitación profesional regular y específica^(28)^.

También es oportuno considerar que las limitaciones de información contravienen las Directrices Curriculares Nacionales (DCN) vigentes para el Curso de Graduación en Enfermería, establecidas por la Resolución n.º 3, de 7 de noviembre de 2001, del Consejo Nacional de Educación^(29)^. Esta reflexión se basa en el hecho de que estas limitaciones constituyen una realidad contraria a lo que orientan las DCN, es decir, la formación de profesionales con competencias y habilidades técnico-científicas, éticas y humanísticas, conscientes de su responsabilidad social, para atender las necesidades de salud individuales y colectivas, con el fin de movilizar transformaciones en la realidad de los grupos humanos.

Sin embargo, también es necesario situar la corresponsabilidad del estudiante en su formación, ya que debe realizar todos los esfuerzos posibles para resolver sus deficiencias de conocimiento y fortalecer sus competencias y habilidades. Con este propósito, la educación superior debe estimular actitudes crítico-reflexivas, a través de procesos emancipadores, que generen autonomía intelectual y, así, movilicen la búsqueda activa del conocimiento^(23)^.

Además, el proceso de enseñanza-aprendizaje debe construirse colectivamente por profesores y estudiantes, por lo que la participación activa de ambos es fundamental e intransferible^(25)^. En este sentido, las relaciones entre los estudiantes son igualmente importantes para establecer redes colaborativas que les ayuden a superar los retos del proceso de enseñanza-aprendizaje^(30)^, como las dificultades en la comprensión de ciertos temas, como la ITBL, y su aplicación coherente en las prácticas de autocuidado y en las prácticas de cuidado dirigidas a grupos humanos.

Como limitación de este estudio, cabe mencionar el hecho de que se llevó a cabo en el curso de grado en enfermería de una universidad pública de Belém, lo que configura un escenario específico, influido por diversos condicionantes, como factores culturales, económicos, políticos, sociales y territoriales. Esto caracteriza una pequeña representatividad geográfica de los datos, teniendo en cuenta que este municipio cuenta con otros cursos de grado en enfermería, sobre todo vinculados a IES privadas, que no se han tenido en cuenta debido a las restricciones impuestas por la pandemia de COVID-19. No obstante, se entiende que las RS aquí reveladas pueden, al menos parcialmente, converger con la realidad vivida/enfrentada por los estudiantes de enfermería de estas y otras IES en los escenarios regional y nacional.

Así, los resultados pueden contribuir a que los estudiantes, profesores y otros actores de la comunidad académica revisen/reestructuren las matrices curriculares y orienten los procesos de enseñanza-aprendizaje en la educación superior, con el fin de mejorar el intercambio de información sobre la ITBL y cualificar la formación académica y profesional. Además, pueden contribuir a que las instancias universitarias competentes y las autoridades públicas reflexionen sobre la necesidad de fortalecer, desde la perspectiva de la intersectorialidad entre los campos de la salud y la educación, las condiciones de bioseguridad en los servicios de salud, con el fin de prevenir casos de ITBL en este público, culminando también en el fortalecimiento de las acciones de control y vigilancia epidemiológica en torno a esta afección.

## CONCLUSIÓN

Este estudio demostró que las RS de los estudiantes de enfermería sobre el intercambio de información sobre la ITBL delinearon dos conjuntos representativos inherentes a sus experiencias con el tema en la educación superior. Dichos conjuntos señalaron limitaciones que debilitaban el intercambio oportuno y la calidad de la información, además de sugerencias que, en opinión de los estudiantes, si se implementaran, podrían resolver las limitaciones o mitigar sus efectos.

Ante estos resultados, cabe destacar que, además de sus contribuciones a la educación en enfermería, la gestión académica en la educación superior y la gestión de los servicios de salud en los que los estudiantes desarrollan actividades prácticas, este estudio puede contribuir a comprender mejor los objetos o fenómenos intersectoriales que involucran la salud y la educación, como el objeto psicosocial investigado aquí.

En este sentido, los resultados tienen el potencial de subsidiar la concepción teórico-metodológica de investigaciones que tengan como objetivo explorar otros aspectos relacionados con la ITBL entre estudiantes de enfermería, valorizando el enfoque cualitativo, sobre todo en su interfaz con la TRS, considerando el potencial de esta teoría para revelar, sistematizar e interpretar subjetividades en el cotidiano de individuos y grupos, razón por la cual ha sido fuertemente utilizada por investigadores de la salud y la educación.

## Data Availability

Los datos que respaldan este estudio están disponibles a solicitud del autor correspondiente.
